# Quantitative characterisation of ipRGCs in retinal degeneration using a computation platform for extracting and reconstructing single neurons in 3D from a multi-colour labeled population

**DOI:** 10.3389/fncel.2022.1009321

**Published:** 2022-11-01

**Authors:** Christopher A. Procyk, Jessica Rodgers, Egor Zindy, Robert J. Lucas, Nina Milosavljevic

**Affiliations:** ^1^Ocular Cell and Gene Therapy Group, Centre for Gene Therapy and Regenerative Medicine, King’s College London, Guy’s Hospital, London, United Kingdom; ^2^Faculty of Biology Medicine and Health, Centre for Biological Timing and Division of Neuroscience, University of Manchester, Manchester, United Kingdom; ^3^Centre for Microscopy and Molecular Imaging, Université Libre de Bruxelles, Brussels, Belgium

**Keywords:** melanopsin, ipRGC, retinal degeneration, Brainbow, segmentation

## Abstract

Light has a profound impact on mammalian physiology and behavior. Intrinsically photosensitive retinal ganglion cells (ipRGCs) express the photopigment melanopsin, rendering them sensitive to light, and are involved in both image-forming vision and non-image forming responses to light such as circadian photo-entrainment and the pupillary light reflex. Following outer photoreceptor degeneration, the death of rod and cone photoreceptors results in global re-modeling of the remnant neural retina. Although ipRGCs can continue signaling light information to the brain even in advanced stages of degeneration, it is unknown if all six morphologically distinct subtypes survive, or how their dendritic architecture may be affected. To answer these questions, we generated a computational platform−BRIAN (Brainbow Analysis of individual Neurons) to analyze Brainbow labeled tissues by allowing objective identification of voxels clusters in Principal Component Space, and their subsequent extraction to produce 3D images of single neurons suitable for analysis with existing tracing technology. We show that BRIAN can efficiently recreate single neurons or individual axonal projections from densely labeled tissue with sufficient anatomical resolution for subtype quantitative classification. We apply this tool to generate quantitative morphological information about ipRGCs in the degenerate retina including soma size, dendritic field size, dendritic complexity, and stratification. Using this information, we were able to identify cells whose characteristics match those reported for all six defined subtypes of ipRGC in the wildtype mouse retina (M1−M6), including the rare and complex M3 and M6 subtypes. This indicates that ipRGCs survive outer retinal degeneration with broadly normal morphology. We additionally describe one cell in the degenerate retina which matches the description of the Gigantic M1 cell in Humans which has not been previously identified in rodent.

## Introduction

A fraction of ganglion cells in the mammalian retina (termed intrinsically photosensitive retinal ganglion cell; ipRGCs) are directly photosensitive thanks to their expression of the photopigment melanopsin (Provencio et al., 2000; Berson et al., 2002; Hattar et al., 2002). In the mouse retina, six morphological subtypes (M1−M6) have been characterized which exhibit differences in dendritic field size, complexity, stratification, electrophysiological properties, and projection targets in the brain (Hattar et al., 2002, 2006; Schmidt and Kofuji, 2009; Berson et al., 2010; Ecker et al., 2010; Schmidt and Kofuji, 2011; Schmidt et al., 2011; Stabio et al., 2018; Quattrochi et al., 2019). This morphological heterogeneity is reflected in the wide array of physiological and behavioral responses to light which ipRGCs mediate, including non-image forming (NIF) responses such as circadian photo-entrainment (Freedman et al., 1999; Berson et al., 2002) and the pupillary light reflex (Lucas et al., 2003). However, more recently they have also been found to support a number of roles in image forming vision including irradiance coding (Brown et al., 2010), irradiance-dependent increases in trial-to-trial reproducibility of visual responses (Storchi et al., 2015), contrast detection (Schmidt et al., 2014), aiding in color opponency (Stabio et al., 2018) and spatial vision (Allen et al., 2017, 2019).

In advanced stages of outer retinal degeneration, the complete loss of rod and cone photoreceptors leaves ipRGCs as the only source of light information which can be relayed to the brain (Brown et al., 2010; Procyk et al., 2015). However, it is still not fully known how ipRGCs are impacted by outer retinal degeneration. Previous reports demonstrate that the total number of ipRGCs develop normally in the *rd*^1^ mouse model of retinal degeneration (Ruggiero et al., 2010), their total number is not significantly affected by the loss of the outer retinal photoreceptors (Semo et al., 2003) and they are generally resistant to retinal injury (Cui et al., 2015; Tran et al., 2019). Antibody labeling has revealed that at least the M1 and M2 subtypes survive with broadly normal retinal anatomy in the *rd1* mouse (Vugler et al., 2008; Lin and Peng, 2013), however it has since been found that a further four subtypes exist which express melanopsin too weakly to be detected by this method (Berson et al., 2010; Ecker et al., 2010). The recent discovery of the M3−M6 subtypes has been facilitated using Tyramide signal amplification (Estevez et al., 2012; Quattrochi et al., 2019) alongside the *Opn4^Cre/+^* mouse line, which demonstrates far superior sensitivity for labeling melanopsin expressing cells and their projections (Brown et al., 2010; Ecker et al., 2010). Therefore, an outstanding question from the literature is whether all six morphologically distinct ipRGC subtypes survive following outer retinal photoreceptor degeneration, and whether they retain normal architecture.

Describing the consequences of retinal degeneration for the ipRGC population requires descriptions of the morphology of individual neurons, a process which is complicated by the degree to which ipRGC dendrites intermingle in the inner plexiform layer. Our approach to this problem was to use the transgenic multicolor labeling technique termed Brainbow (Cai et al., 2013) which uses Cre-loxP recombination to drive stochastic expression of up to four reporter proteins in individual neurons, producing tens of hues across the targeted population (Livet et al., 2007; Cai et al., 2013). The resultant cell-to-cell variation in abundance of the various reporter proteins may allow individual cells to be identified by their unique profile of fluorescence across various wavelengths (usually depicted and analyzed as pseudocolor). Brainbow, and related multi-color labeling approaches, have been applied to a variety of neuronal and non-neuronal tissues in diverse species (Hampel et al., 2011; Pan et al., 2013; Robles et al., 2013; Hammer et al., 2015; Nern et al., 2015; Wang et al., 2017; Sakaguchi et al., 2018). However, in applying this approach we found that optimal analysis of multi-label images has remained a major technical challenge. The most technologically straightforward approach is for a human user to reconstruct individual cells (or parts thereof) by manually linking contiguous image elements with a common “color” (as a proxy for a common ratio of expression for the different fluorophores) (Lakadamyali et al., 2012; Robles et al., 2013; Hammer et al., 2015; Sakaguchi et al., 2018). This method becomes increasingly unwieldy when working in 3D, and with cells with a complex morphology, and intermingled processes such as ipRGCs in the retina and their projections in the brain. A recent improvement of this technique has applied computational refinements to the user input in order to improve the reliability of such manual segmentation and simplified the challenge of linking elements in a 3D volume by allowing the user to view the image as sequential 2D image stacks (Roossien et al., 2019). However, as structure becomes much more apparent when viewing in full 3D for many cells, one may expect the simplification to 2D stacks to have a cost in terms of accuracy and productivity.

Here, we design a novel multi-color image analysis platform, which we term (BRIAN; BRaInbow Analysis of individual Neurons), that accepts multi-color images, applies an initial image-filtering step to exclude uninformative voxels, before allowing user-guided identification of voxel clusters in Principal Component (PC) space to be used to generate simplified images comprising voxels of similar color suitable for individual tracing in full 3D and recombination to generate a composite image of all traced elements within the region of interest. We first validate this method using artificial images and images of structurally simpler bipolar cells, before continuing to demonstrate its utility to reconstruct images of individual ipRGCs from the retina of degenerate *Opn4*^Cre/+^*;rd/rd* mice transfected with Brainbow virus. Following reconstruction of their 3D morphology, we compare the soma sizes and the extent, complexity, and location of dendritic fields within the inner-plexiform layer with previous descriptions of ipRGC anatomy in the wildtype retina. We find ipRGCs whose characteristics match those reported for all six subtypes of ipRGC in the wildtype retina (M1−M6). This indicates that ipRGCs survive outer retinal degeneration, at least at the anatomical level with broadly normal morphology. We finally demonstrate that BRIAN can be applied to trace ipRGC projections with single cell resolution in 3D in several demanding applications including for complex and heavily intertwined processes that travel long distances in both the SCN and dLGN, respectively.

## Results

We set out to determine the extent to which ipRGCs remain morphologically intact in advanced degeneration. The Brainbow multi-labeling approach represents an attractive option for addressing that problem as: (1) there is a well-defined transgenic mouse line (*Opn4^Cre^*) enabling reporter gene expression in these cells (Hattar et al., 2006); (2) separation of the 6 morphologically distinct ipRGC subtypes requires detailed 3D anatomical reconstruction; and (3) ipRGCs have wide-ranging and interleaved processes, making them promising candidates for the potential increases in tracing efficiency afforded by multi-colored labeling approaches.

**FIGURE 1:**
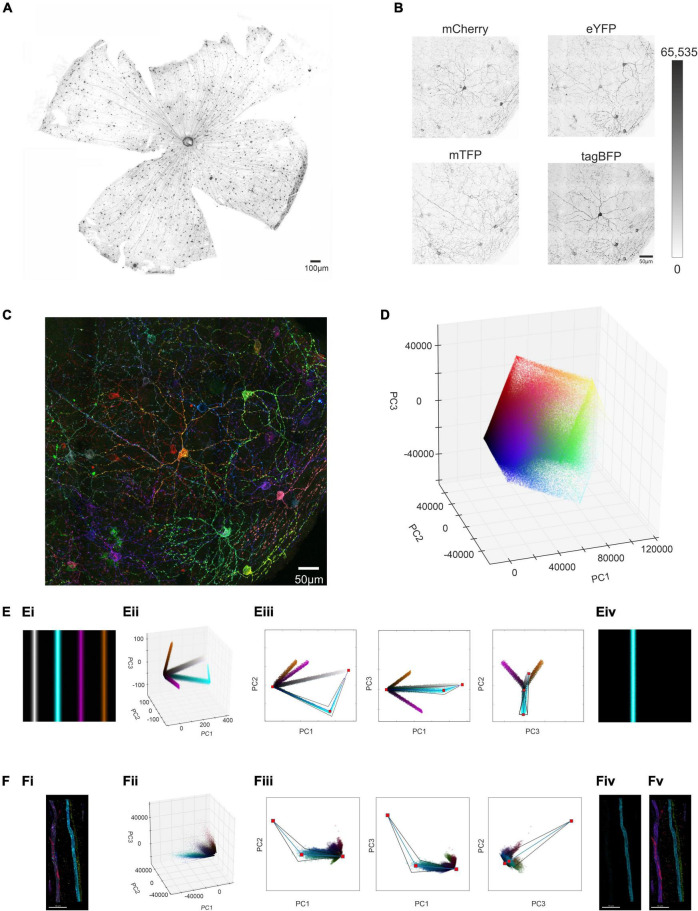
Identifying and isolating voxel clusters from multi-color images in Principal Component Space. **(A)** Following a unilateral intravitreal injection of Brainbow viruses into an *Opn4^Cre/+^* mouse, immunohistochemical labeling against the mCherry reporter protein (in monochrome) demonstrates strong labeling of cells distributed across the retinal ganglion cell layer. **(B)** Maximum projection of the immunohistochemically labeled mCherry, mTFP eYFP and tagBFP reporter proteins from a region of interest (ROI) from an *Opn4^Cre/+^* retina demonstrates variable reporter protein expression between individual intrinsically photosensitive retinal ganglion cells (ipRGCs) in the *Opn4^Cre/+^* retina. (Note weak expression of tagBFP and high background noise) **(C)** Representative confocal image of the ROI shows stochastic labeling of individual cells when the combined maximal projection of the three individually recorded channels mCherry (pseudocolored Red), eYFP (pseudocolored Green) and mTFP (pseudocolored Blue) from panel **(B)**, following immunohistochemical labeling. **(D)** Principal component analysis (PCA) conducted on the intensity values of the constituent voxels of ROI in panel **(B)** were plotted in a 3D representation of PC space (PC1 vs. PC2 vs. PC3). **(E,Ei)** Simple simulated image of four colored bars, with hue and saturation kept constant across each bar, but with lightness varying from 0 (black) at the outer edge to either 0.6 (in the case of purple and orange) or 0.9 (in the case of cyan and gray) in the center (see online methods and Supplementary Figure 1 for further explanation). **(Eii)** 3D representation of PC space from the image in panel **(Ei)** shows that the cyan cluster of voxels diverging from the black point, reaches a point of inflection, and tends toward the white point. **(Eiii)** Extraction of the cyan cluster (from black point to the white point via the inflection point) in 3 × 2D representations of PC space (PC1 vs PC2, PC1 vs. PC3 and PC2 vs. PC3) reconstructs the entirety of the cyan bar **(Eiv)**. For more details see Supplementary Figure 1 and codes are available for this Figure at: https://github.com/lucasgroup/BRIAN (BBStatic.py) **(F,Fi)** Maximum projection of a ROI of an optic nerve from *Opn4^Cre/+^;rd/rd* mouse unilaterally intravitreally injected with the AAV Brainbow virus, following immunohistochemical labeling for mCherry (pseudocolored red), eYFP (pseudocolored green) and mTFP (pseudocolored blue) reporter proteins shows four fibers. **(Fii)** 3D representation of PC space from the image in panel **(Fi)** shows similar behavior as the simulated image in panel **(Ei)**. **(Fiii)** Extraction of the cyan cluster (from black point to the white point via the inflection point) reconstructs the cyan fiber **(Fiv)**. **(Fv)** The same approach was applied to four other identifiable clusters of pixels and enough of the voxels from each cluster were extracted for all four of the fibers from the optic nerve in **(Fi)**.

To trial this approach, we first applied the Brainbow virus combination to a visually intact *Opn4^Cre^* mouse by intravitreal injection, collected the retina 4 weeks post injection, and subjected it to immunocytochemistry for the reporter proteins using spectrally distinct fluorescent secondary antibodies (Figure 1A). We employed confocal microscopy to capture 3D images of immunofluorescence corresponding to the 4 reporter proteins over an ROI from an *en face* view of the injected retina (Figure 1B). We found that the signal for tagBFP was much lower than for the other 3 reporter proteins (note high background signal in Figure 1B; see section “Methods”) and excluded this channel from further analysis. Images corresponding to fluorescence for the three remaining reporter proteins were captured at 16-bit resolution. Stochastic variation in expression of the 3 proteins (mCherry, eYFP, and mTFP) between individual ipRGCs was apparent as differences in brightness for given voxels across the 3 images (Figure 1B) and in a pseudocolored representation of the region of interest (ROI) in which mCherry, eYFP, and mTFP signals were assigned to red, green, and blue subpixels respectively (Figure 1C).

### Principal component analysis to identify voxel clusters

We set out next to develop a method for reliably segmenting the ROI in Figure 1C. To minimize the time taken for manual or semi-automated tracing of individual ipRGCs (which can have complex and extensive dendritic trees), we aimed first to produce simplified versions of the image containing voxels from a single cell. To this end, we set out to identify voxel clusters sharing a similar ratio of immunofluorescence across the three reporter proteins. To achieve an optimal representation of the variation in reporter expression across the population of voxels within any given ROI, we applied principal component analysis (PCA) to immunofluorescence intensity measures for mCherry, eYFP and mTFP, and visualized the outcome as a scatter plot of the voxels in the first 3 principal components (Figure 1D). Voxels with similar color tended to appear together in principle component space. However, there were not clear clusters as would be expected if voxels from individual cells shared common ratios of reporter protein expression. To understand that voxel distribution pattern we turned to a simple synthetic image (Figure 1Ei), comprising 4 lines differing in hue and saturation (between lines) and lightness (within each line) to recreate the situation in our ROI. The distribution of voxels from this simple image in principle component space is shown in Figure 1Eii and Supplementary Figures 1A–C. Voxels from each line in the PC plot of this synthetic image could fall around vectors diverging from the black point (Figure 1Eii and Supplementary Figures 1A–C), and for lines with higher brightness the white point, and meeting at an “elbow point” (Supplementary Figures 1D,E).

We then developed a method for separating clusters based upon this fundamental pattern. Thus, a graphical user interface (GUI) was generated in which the PC distribution was presented in 3 × 2D projections (Figure 1Eiii). A user could then view this distribution and manually place an “elbow” marker in 2 of the 3 PCA plots that, when linked to black and white points, defined the vectors around which a cluster formed. A tapered polygon was then fitted around these lines whose width was adjusted by the user to capture most of the cluster of interest (Figure 1Eiii and Supplementary Figures 1F–H). The resultant polygon was then applied as a mathematical filter to the original image to extract pixels comprising a single cluster, to successfully isolate single image features (Figure 1Eiv). Further explanation for the design of the extraction template is provided in methods and Supplementary Figure 1). Analysis code for the interactive tool to explore clusters in these simulated images is available at https://github.com/lucasgroup/BRIAN (BBStatic.py).

To determine whether the method for cluster extraction developed for the synthetic image was suitable for real biological samples, we turned first to a sample in which we could easily relate the distribution of voxel clusters in PC space to image content. Transducing ipRGCs in visually intact *Opn4^Cre/+^* retinas with Brainbow virus resulted in sparse labeling of axons in the optic nerve, allowing individual fibers to be readily identifiable in transverse sections of this tissue (Figure 1Fi). Voxels from this representative ROI in the optic nerve containing only four fibers formed a qualitatively similar distribution in PC space to that observed in the synthetic image, with vectors appearing to diverge from the calculated black point (and to a lesser extent converging on the white) (Figure 1Fii). A plausible explanation for this distribution is that thresholding and saturation effects in image acquisition produce variations in fluorescence ratio across voxels differing in brightness. Applying the polygon voxel extraction approach successfully segmented this cropped image to produce separate images of each fiber (Figure 1Fiii). While very bright (and likely very dim) voxels were lost in cluster extraction, the form of each of the four fibers was substantially retained in the segmented images confirming the fundamental suitability of the approach (Figures 1Fiv,v).

**FIGURE 2:**
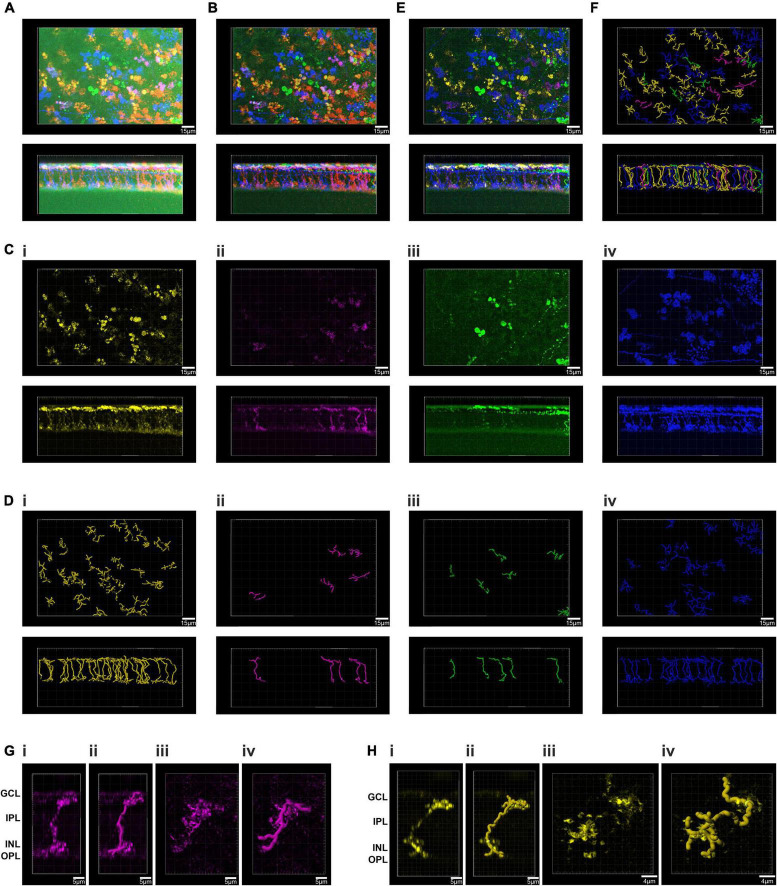
Principal component analysis and spatial reconstruction of bipolar cells in the GRM6^Cre/+^ retina. **(A)** Confocal tile-scanned image of a representative wholemount retina from an Grm6Cre/ +; mouse unilaterally intravitreally injected with the AAV Brainbow virus, following immunohistochemical labeling for mCherry (pseudocolored red), eYFP (pseudocolored green) and mTFP (pseudocolored blue) reporter proteins in *en face* (top) and Z (bottom) views. **(B)** The ROI from panel **(A)** following pre-filtering. **(C)** Spatial reconstruction of voxels from the four isolated clusters **(Ci–iv)** in an *en face* (top) and Z (bottom) views. **(D)** Filament tracer reconstructions of the four clusters isolated using the Brainbow analysis of individual neurons (BRIAN) platform **(Di–iv)** in an *en face* (top) and Z (bottom) views. **(E)** Spatial reconstruction of voxels from the four isolated clusters in an *en face* (top) and Z (bottom) views. **(F)** Filament tracer reconstructions of all four clusters isolated using the BRIAN platform an *en face* (top) and Z (bottom) views revealed 67 single cells, out of 76 in the starting image **(A)**. **(G)** Close up of a representative rod bipolar cell. Spatial reconstruction of voxels from one rod bipolar cell in Z **(Gi)** and XY **(Giii)** views. Filament tracer reconstruction of the rod bipolar cell in Z **(Gii)** and XY **(Giv)** views. **(H)** Close up of a representative cone bipolar cell. Spatial reconstruction of voxels from one cone bipolar cell in Z **(Hi)** and XY **(Hiii)** views. Filament tracer reconstruction of the cone bipolar cell in Z **(Hii)** and XY **(Hiv)** views.

We next looked to test our approach at segmenting multi-color images on a retinal cell population with less anatomical complexity than presented by ipRGC dendritic trees. To this end, we injected the Brainbow virus combination to the eye of a visually intact mouse (*Grm6^Cre/+^*) in which Cre recombinase is expressed in the much more numerous ON bipolar cells (Morgans et al., 2009). We harvested the retina of the *Grm6^Cre/+^* mouse 4 weeks post injection and subjected it to immunocytochemistry and imaging as previously described (Figure 2A). To focus the PC analysis on the most informative voxels, we then applied an image filtering step (see methods) that eliminated high frequency noise and voxels with low brightness (Figure 2B). We then applied the PC analysis to an ROI and used the GUI to define clusters with similar color (Figure 2C). When the voxels falling within these clusters were extracted and viewed in their location in the original image (Figures 2Ci–iv, it was clear that each cluster contained voxels from more than one cell. Nevertheless, the degree of image segmentation achievable with this method was sufficient to individual cells to be readily resolved (Figures 2Ci–iv. Accordingly, resultant images could be used to trace and reconstruct single cells in 3D (Figures 2Di–iv with a total of 67 fluorescent cells reconstructed in this ROI (Figures 2E,F). The quality of these reconstructed images was sufficient to identify the two major sub-division of ON bipolar cell (close-up Figures 2G,H). Thus, one group had features characteristic of rod bipolar cells: “bulbous” or “granular” terminals, without much branching, stratifying in or near the ganglion cell layer, and a relatively thick axon arborizing over a relatively small area (Figures 2Gi–iv and Supplementary Video 1; Tsukamoto and Omi, 2017). The other group had less granular or bulbous axon terminals, dendrites covering a wider area with more branching, as expected for ON cone bipolar cells (Figures 2Hi–iv and Supplementary Video 2; Tsukamoto and Omi, 2017).

### Development of BRIAN using ipRGCs in the *Opn4^Cre/+^* retina

**FIGURE 3:**
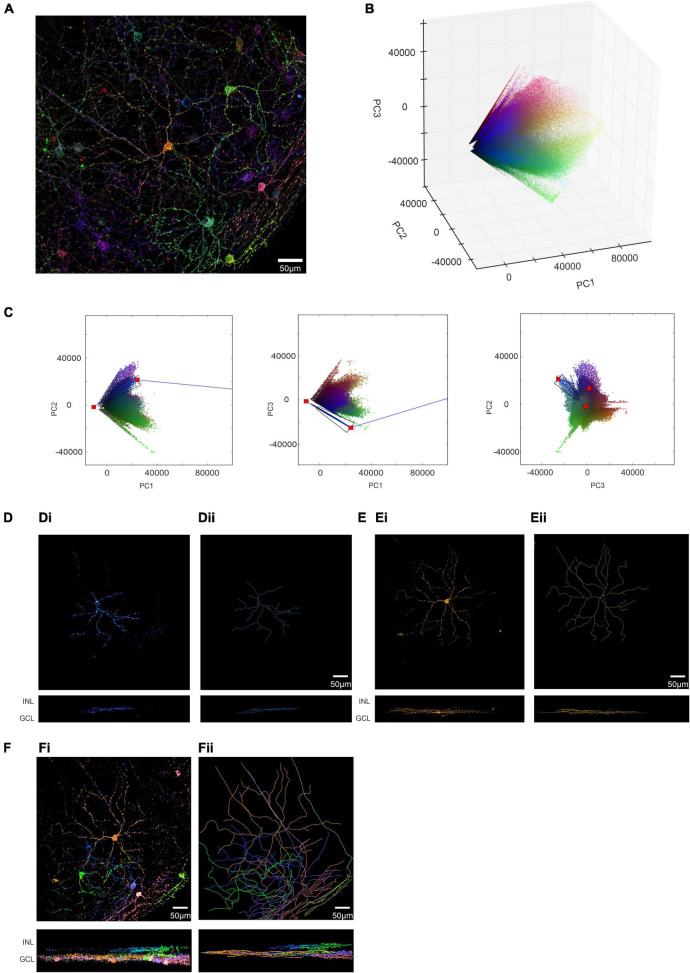
Development of BRIAN and PCA for single cell isolation and 3D spatial reconstruction in the *Opn4^Cre/+^* retina. **(A)** Pseudocolored immunofluorescence image of the ROI in Figure 1C following image pre-filtering to reduce background noise and remove voxels with signal in only one channel (see Online Methods). **(B)** 3D representation of PC space (PC1 vs. PC2 vs. PC3) of the ROI from the *Opn4^Cre/+^* retina following image pre-filtering. **(C)** Following pre-filtering of the ROI an identifiable “blue” cluster of voxels became apparent in PCA space, shown here in sequential 2D projections in PC space of randomly selected 80,000 voxels (a number which we found to give a good visual approximation of the clusters at reduced rendering time). Blue lines depict the center of the vector directions of this cluster in each 2D plot from black and white points; red dots show origins (black and white points) and intersection of these vectors. A user defined trapezoid encompassing the voxels in this cluster is shown in black. **(D)** Spatial location of voxels in XY (upper) and XZ (lower) orientations of voxels from the ROI falling within the region of PCA space described by the trapezoid in panel **(C)** project an image consistent with that of a single cell in XY and XZ dimensions **(Di)** and its reconstruction **(Dii)**. **(E)** The same process as in panels **(C,D)** was applied to an identifiable “orange” cluster in PCA space (shown in **C**) to generate an image consistent with that of a single cell in XY and XZ dimensions **(Ei)** and its reconstruction **(Eii)**. **(F)** Spatial location of voxels in XY (upper) and XZ (lower) orientations from 6 separate clusters (color of each voxel matches that of its appearance in the pseudocolored representation of this ROI in panel **(A)** isolated from the PCA **(Fi)**. **(Fii)** Filament tracer images of the six voxel clusters in panel **(C)** to reconstruct morphology of 6 differently colored cells within the ROI in *en face* (upper) and Z (lower) projections. Locations of inner nuclear layer (INL) and ganglion cell layer (GCL) provided as references for Z-projection images.

We next applied our GUI to the more demanding problem of separating voxel clusters from the more anatomically complex dendritic fields of ipRGCs in the ROI from a visually intact *Opn4^Cre/+^* retina (Figure 1C). Following image filtering (as above), we further simplified the image by removing voxels with significant signal in only 1 fluorescence channel to confirm that the image segmentation process could separate cells with multi-reporter labeling. The filtered individual channels from our ROI (see methods; Supplementary Figures 2A–D) were then combined as three representations of the image with mCherry (pseudocolored red), eYFP (pseudocolored green) and mTFP (pseudocolored blue) and demonstrated the stochastic labeling of individual ipRGCs (Figure 3A and Supplementary Figures 2B,E).

We applied PCA to the immunofluorescence intensity measures for our newly filtered mCherry, eYFP and mTFP channels and visualized clear voxel clusters with vectors diverging from the calculated black point, and in some cases converging on the white point (Figure 3B) which were not clearly apparent in the unfiltered PCA plot (Figure 1D and Supplementary Figures 2C,F). In this instance, we found that including two other cues in our GUI was helpful in identifying clusters. Firstly, the symbol for each voxel in PCA space was plotted using the same color as that voxel in a pseudocolored representation of the original ROI. Secondly, the GUI included a live display of the location of chosen voxels in a 2D projection of the ROI, allowing the user to refine the extraction polygon with reference to a simplified form of the segmented image (see Supplementary Figure 2G). Using this approach, it was possible to identify 6 clusters in PC space from this representative ROI (shown for 1 representative cluster in Figure 3C). As the voxels subjected to PCA retained indexing to their location in X, Y and Z dimensions in the original input image, applying mathematical filters based upon the PC clusters segmented the starting region of interest into separate 3D images enriched for voxels from a single cell (shown for 2 representative clusters in Figures 3Di,Ei). These could be viewed as a composite image, revealing the spatial relationship between the six extracted cells in 3D (Figure 3Fi). The separate images were also suitable for 3D tracing of individual cells, including dendritic architecture, using the commercially available “Filament Tracer” plugin to Imaris (Bitplane, Zurich; Figures 3Dii,Eii). Applying this tracing in 3D enabled visualization of three critical features of ganglion cell morphology for each neuron: the complexity and size of dendritic tree in *en face* view; and the depth in the inner plexiform layer (IPL) at which their dendrites arborize (Figure 3Fii).

**FIGURE 4:**
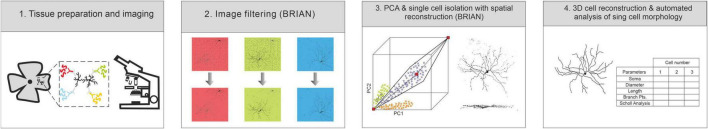
Schematic of the main elements of the BRIAN platform: **(1)** Tissue preparation and imaging: **(2)** image pre-filtering and preparation [to remove uninformative voxels, including those with signal for only a single fluorophore (blue panel)], **(3)** PCA and single cell identification, and **(4)** 3D spatial reconstruction with automated single cell data extraction.

The steps required to achieve effective single cell anatomical reconstruction of the Brainbow labeled tissue are presented in schematic form in Figure 4, and together comprise an analysis process which we term BRIAN. Following The key steps of the analysis platform are: (1) tissue preparation and imaging; (2) image filtering; (3) segmentation of the image based upon identification of voxel clusters following PCA of fluorescence intensity for each fluorophore; and (4) 3D reconstruction of single cell morphology suitable for automated analysis.

### Quantitative morphological analysis of individual ipRGCs in the degenerate retina using BRIAN

**FIGURE 5:**
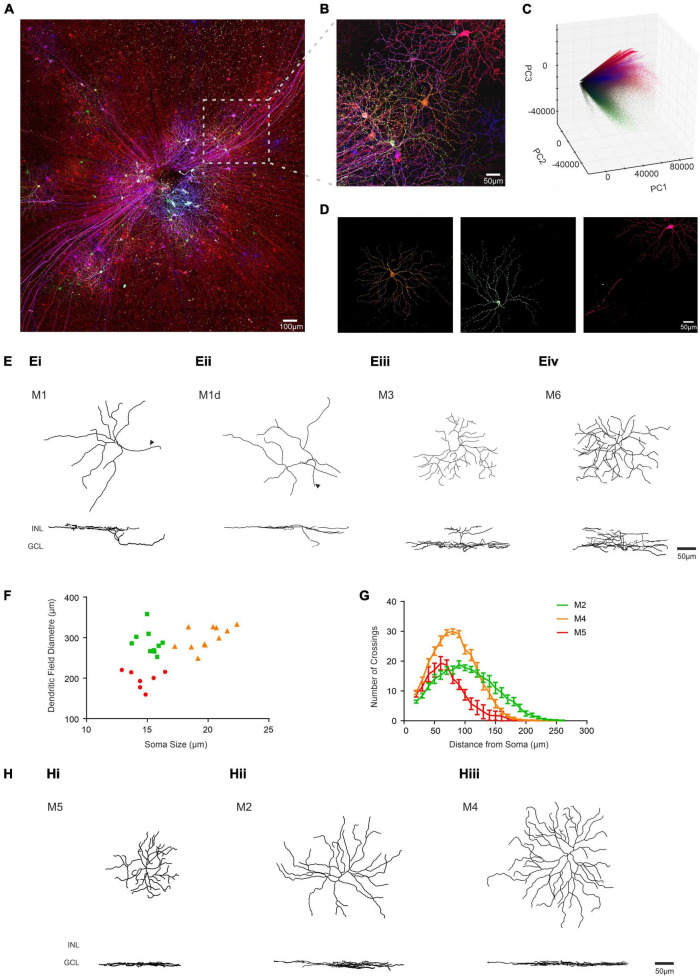
Identifying ipRGC subtypes in the degenerate retina through quantitative morphological characteristics obtained using BRIAN. **(A)** Maximum projection of a representative wholemount retina from an *Opn4^Cre/+^;rd/rd* mouse unilaterally intravitreally injected with the AAV Brainbow virus, following immunohistochemical labeling for mCherry (pseudocolored red), eYFP (pseudocolored green) and mTFP (pseudocolored blue) reporter proteins. **(B)** Representative filtered ROI from the boxed region in panel **(A)** shows dense labeling of Retinal Ganglion cells with considerable variation in immunofluorescence for the three reporter genes. **(C)** 3D representation of PC space (PC1 vs. PC2 vs. PC3) for all the voxels from the filtered ROI shown in panel **(B)** from which distinct clusters can be observed diverging from the “black point.” **(D)** *En face* views of the spatial distribution of voxels assigned to each of three distinct clusters in PC space from the ROI in panel **(B)** reveal the shape of 3 distinct cells. Color of voxels in each cluster (orange, green and pink) matches their appearance in the pseudocolored image in panel **(B)**. **(E)** Filament tracer reconstructions of 4 single ipRGCs isolated using the BRIAN platform from *Opn4^Cre/+^;rd/rd* retinas in *en face* (top) and Z (bottom) views representative of M1 **(Ei)**, M1d **(Eii)**, M3 **(Eiii)**, and M6 **(Eiv)** subtypes. Note location of dendrites in outer portion of IPL (close to INL) for M1 cells and in both upper and lower IPL for M3 and M6; and soma displaced in the INL for the M1d (arrows denote axons). **(F)** Soma size and dendritic field diameter showed substantial variation across ipRGCs isolated using this method and stratifying exclusively in the inner sublamina of the IPL (putative M2, M4, and M5 cells appear as green, orange, and red symbols respectively); as did dendritic complexity as determined by Sholl analysis (**G**; color code as for panel **F**). **(H)** Filament tracer reconstructions in both an *en face* (upper) and Z (lower) view of 3 ipRGCs isolated using the BRIAN platform representative of M5 **(Hi)**, M2 **(Hii)**, and M4 **(Hiii)** subtypes. Locations of inner nuclear layer (INL) and ganglion cell layer (GCL) provided as references for Z-projection images.

We next applied the BRIAN process to our primary scientific objective, describing the morphology of ipRGCs in advanced retinal degeneration. Intravitreal injection of the “Brainbow” viruses to an *Opn4^Cre/+^* mouse homozygous for the *Pde6b^rd^*^1^ mutation (Pittler and Baehr, 1991; Chang et al., 2002) (designated here *Opn4*^Cre/+^*;rd/rd* mice), which causes aggressive and near complete loss of rod and cone photoreceptors, resulted in divergent reporter gene expression across the population of retinal ganglion cells (shown for representative tile-scan of a retina in Figure 5A). Regions of strong expression in this tissue were suitable for segmentation using the PCA approach (shown for a representative region in Figures 5B–D). To confirm that this strategy was suitable for different samples we applied it to ROIs across 4 retinas to isolate images of 35 ipRGCs. We then asked whether these images were suitable for quantitative morphological characterization. Segmented images were traced and analyzed using Filament Tracer, recording soma size and the location, size, and complexity of dendritic fields (critical parameters for distinguishing among ipRGC subtypes) (Hattar et al., 2002; Schmidt and Kofuji, 2009; Berson et al., 2010). Importantly, anatomical features of these reconstructed cells were sufficiently accurate and detailed for us to unambiguously allocate most to one of the six ipRGC subtypes thanks to their similarity to published descriptions from visually intact mice acquired using single-cell or sparse labeling approaches (Schmidt and Kofuji, 2009, 2011; Estevez et al., 2012; Quattrochi et al., 2019). Four reconstructed cells had dendrites in the inner (OFF) sublayers of the IPL, characteristic of the M1-type ipRGC. This population had an average soma size of 12.57 ± 0.82 μm (mean ± SEM) and an average dendritic field size of 227.21 ± 7.41 μm (mean ± SEM; Figure 5Ei). One of these cells had its soma in the inner nuclear layer matching the known description of displaced M1-type cells (dM1; Figure 5Eii). We also found an example of a cell stratifying in both ON and OFF sublaminae of the IPL, identifying it as a member of the rare M3-type ipRGC (dendritic field size = 228.12 μm; soma size 18.5 μm; total branch points = 44; Figure 5Eiii). We additionally identified one cell whose dendrites were bistratified in both the ON and OFF sublaminae of the IPL, but regularly crossed between the two, a hallmark feature of the M6 subtype (dendritic field size = 239 μm; soma size 13.9 μm; total branch points = 41; Figure 5Eiv). All other known ipRGC subtypes exclusively stratify in the outer (ON) sublaminae of the IPL. 28 of the ipRGCs extracted from the *Opn4*^Cre/+^*;rd/rd* retina had this morphology. Seven of these cells with smallest dendritic field (mean ± SEM = 197.17 ± 8.4 μm) also had a small soma (mean ± SEM = 14.60 ± 0.44 μm) and high dendritic complexity (35.43 ± 1.9 branch points) matching the description of the M5 subtype (Ecker et al., 2010; Figures 5F(Hi). M2 and M4 ipRGCs are significantly harder to distinguish as they exhibit overlap in dendritic field size. However, M4 cells have on average larger soma sizes and slightly larger and more complex dendritic architecture (Estevez et al., 2012). Separating the population according to both soma size and number of dendritic branch points allowed separation into 2 groups whose members did not overlap in either dimension. The 11 cells with the largest soma size and most branch points are likely members of the M4-type and the remaining ten, M2s (Figures 5Hii,iii, Table 1, and Supplementary Table 1). Indeed, Sholl analysis confirmed that the M4 group identified in this way and had the expected increase in dendritic complexity (Figure 5G). A representative reconstructed cell from each of the six identified subtypes is shown in both X, Y and X, Z orientations in Figures 5Ei–iii,Hi–iii. The descriptive statistics for this analyzed population are described in Table 1 and Supplementary Table 1 where they are compared to published values for ipRGCs in the intact mouse retina.

**TABLE 1:** Quantitative morphological analysis of ipRGCs in the *Opn4^Cre/+^*; *rd/rd* mouse using BRIAN. **(A)** Population data (Mean ± SEM) for stratification, soma size, dendritic field diameter and total number of branch points for the six reported ipRGC subtypes identified from four *Opn4^Cre/+^*; *rd/rd* retinas using the BRIAN platform. Single cell data was collected using the commercially available plug-in Filament Tracer (IMARIS, Bitplane). Visually intact data collected from published literature on *Opn4^Cre/+^* mice and previously reported.

Subtype	Model	Stratification	Soma size (pm)	Dendritic field diametre (μm)	Number of branch points	Fraction of total	
**Ml**	Retinally Degenerate	OFF	12.6 ± 0.8	227.2 ± 7.4	8.3 ± 1.3	4/35	
	Visually Intact		13.9 ± 0.5	290.1 ± 16.5	10.2 ± 1.6	–	1
**M2**	Retinally Degenerate	ON	15.2 ± 0.3	287.6 ± 9.6	27.2 ± 2.8	10/35	
	Visually Intact		15.7 ± 0.4	316.6 ± 13.8	24.4 ± 1.5	–	1
**M3**	Retinally Degenerate	Bistratified	18.5	228.1	44.0	1/35	
	Visually Intact		17.8 ± 0.6	477.4 ± 20.1	Not Reported	–	2
**M4**	Retinally Degenerate	ON	19.9 ± 0.5	298.6 ± 8.3	45.5 ± 1.9	11/35	
	Visually Intact		21.0 ± 0.4	359.6 ± 12.8	38.2 ± 1.6	–	1
**M5**	Retinally Degenerate	ON	14.6 ± 0.4	197.2 ± 8.4	35.4 ± 1.9	7/35	
	Visually Intact		14.2 ± 2.4	223.7 ± 43.9	52.1 ± 12.5	–	3
**M6**	Retinally Degenerate	Bistratified	13.9	239	41	1/35	
	Visually Intact		12.7 ± 1.8	216 ± 30	100 ± 27	–	4
**Uncategorised**	Retinally Degenerate	OFF	13.8	546	21	1/35	

^1^Ecker et al. (2010) Melanopsin-expressing retinal ganglion cell photoreceptors: Cellular diversity and role in pattern vision.

^2^Schmidt and Kofuji (2011) Structure and function of the bristratified intrinsically photosensntive ganglion cells in the mouse.

^3^Stabio et al. (2018) The M5 Cell: A color-opponent intrinsically photosensitive retinal ganglion cell.

^4^Quattrochi et al. (2019) The M6 cell: A small-field bistratified photosensitive retinal ganglion cell.

Although almost all cells reconstructed from *rd/rd* retinas could thus be assigned to one of the known ipRGC types with reasonable confidence, there was one for which this was not the case. This neuron stratified in the OFF sublamina of the IPL, with a relatively small oval shaped soma (13.8 μm) and simple dendritic complexity (21 branch points), indicating it would belong to the M1 subtype (Supplementary Figure 3). However, its dendritic field size was nearly twice the diameter of other M1 ipRGCs (546 μm) identified using BRIAN (Table 1 and Supplementary Table 1).

### Tracing individual ipRGC projections in the brain

**FIGURE 6:**
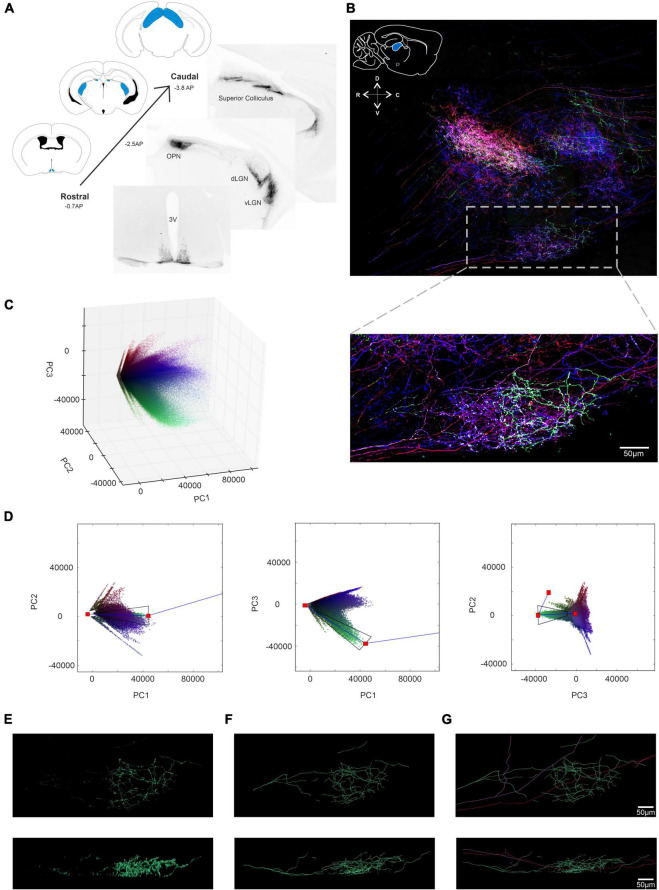
Using BRIAN to trace axonal projections of ipRGCs in the Lateral Geniculate Nucleus (LGN). **(A)** Coronal Sections from an *Opn4^Cre/+^;rd/rd* mouse brain following intravitreal AAV Brainbow injection. Immunohistochemical labeling for the mCherry reporter protein in monochrome reveals strong projections to regions including the SCN (suprachiasmatic Nucleus), OPN (Olivary Pretectal Nucleus), the dorsal and ventral LGN (Lateral Geniculate Nucleus) and the SC (Superior Colliculus). Schematic diagrams from the mouse atlas illustrate the densely stained regions in blue. **(B)** (Top) Confocal tile-scanned image of a 100 μm thick sagittal section of the *Opn4^Cre/+^;rd/rd* dLGN following a unilateral intravitreal AAV Brainbow injection and immunohistochemical staining for the mCherry (pseudocolored red), eYFP (pseudocolored green) and mTFP (pseudocolored blue) reporter proteins. Inset: schematic diagram of a sagittal cross-section from the mouse atlas (above) illustrates anatomical position of the dLGN in blue. ROI from the boxed region following pre-filtering with BRIAN. **(C)** 3D representation of the PC space (PC1 vs. PC2 vs. PC3) for the filtered ROI shown in panel **(B)** which depicts all voxels in the filtered image. **(D)** 3 × 2D representations of the PCA space (from left to right; PC1 vs. PC2, PC1 vs. PC3 and PC2 vs. PC3) and the extraction polygon used to isolate the turquoise cluster. **(E)** Spatial reconstruction of voxels from the isolated turquoise cluster in panel **(D)** in both an *en face* (upper) and Z (lower) view. **(F)** Filament tracer reconstructions in both an *en face* (upper) and Z (lower) view of the turquoise cell isolated using the BRIAN platform. **(G)** Filament tracer reconstructions for the three single cell axonal projections (turquoise, pink and purple) combined in (top) XY and (bottom) XZ dimensions (Franklin and Paxinos, 2007).

Having applied BRIAN to our primary scientific problem of reconstructing the complex ipRGC dendritic fields in retinally degenerate mice, we finally wondered whether it could be used for an even more demanding application – reconstructing ipRGC axonal projections across large brain volumes. As ipRGCs send their axons to the brain, we were able to use a brain collected from *Opn4^Cre/+^*;*rd/rd* mice receiving unilateral intravitreal injection of “Brainbow” viruses for this purpose (Figure 6A). We first concentrated on the main thalamic visual center, the dorsal lateral geniculate nucleus (dLGN) (Brown et al., 2010; Ecker et al., 2010; Figure 6B). We identified a 495 μm × 206 μm × 100 μm ROI in the sagittal plane of the dLGN contralateral to the injected eye that contained numerous immunofluorescence axons exhibiting high color and brightness variation (Figure 6B; inset). We filtered the resulting image using the same approach as applied in the retina and ran this region through our 3D PCA (Figure 6C). We were able to use the tapered polygon template to isolate three voxel clusters with good confidence, that appeared turquoise, pink, and purple in the pseudocolored representation of the image in principal component space (shown for the turquoise cluster in Figure 6D). Following extraction (Figure 6E) and 3D recreation (Figure 6F), these clusters were processed with Filament Tracer to reveal the axonal projections of 3 neurons (Figure 6G). To assess the suitability of these reconstructions for quantitative analysis, we turned to the most complex of the 3 axonal projections (Figure 6F). We were able to measure the total length (3,278.37 μm) and number of branch points (65) for this “turquoise” filament.

**FIGURE 7:**
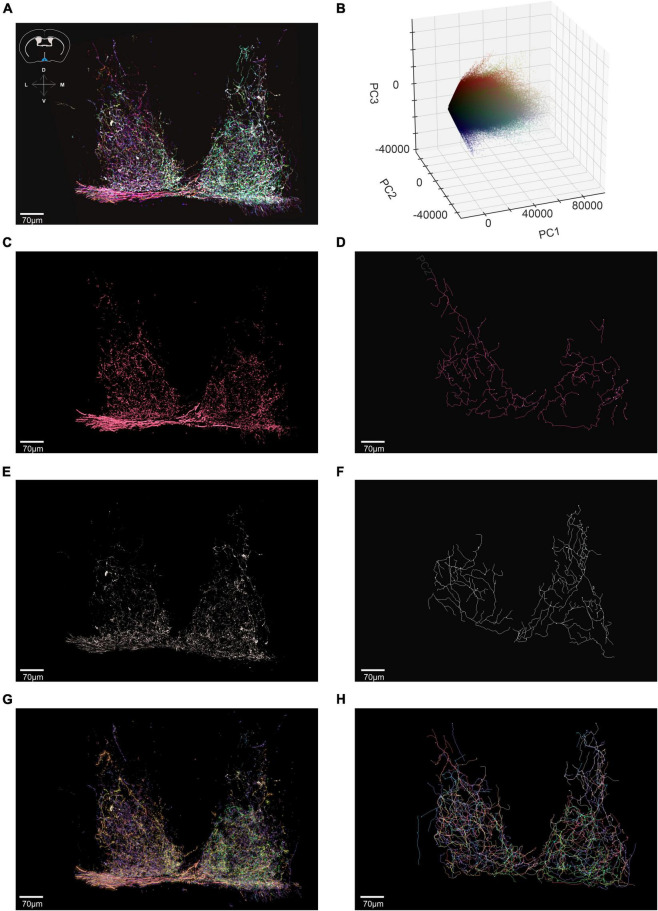
Using BRIAN to trace axonal projections of ipRGCs in the Suprachiasmatic Nucleus (SCN). **(A)** Confocal tile-scanned image of a representative of a 60 μm thick coronal section of the *Opn4^Cre/+^;rd/rd* SCN (Suprachiasmatic Nucleus), following a unilateral intravitreal AAV Brainbow injection and immunohistochemical staining for the mCherry (pseudocolored red), eYFP (pseudocolored green) and mTFP (pseudocolored blue) reporter proteins following pre-filtering. Inset: schematic diagram of a coronal cross-section from the mouse atlas (above) illustrates anatomical position of the SCN in blue. **(B)** 3D representation of the PC space (PC1 vs. PC2 vs. PC3) for the filtered ROI shown in panel **(A)** which depicts all voxels in the filtered image. **(C)** Spatial reconstruction of voxels from the isolated red cluster in an *en face* view. **(D)** Filament tracer reconstructions in an *en face* view of the red cluster isolated using the BRIAN platform revealed 3 continuous filaments (which may represent the axonal projections of 3 cells with similar color or parts of the same neuron that would be continuous if viewed in a larger brain volume). **(E)** Spatial reconstruction of voxels from the isolated white cluster in an *en face* view. **(F)** Filament tracer reconstructions in an *en face* view of the white cluster isolated using the BRIAN platform. **(G)** Spatial location of voxels in *en face* view from 7 separate clusters (color of each voxel matches that of its appearance in the pseudocolored representation of this ROI in **A**) isolated from the PCA **(B)**. **(H)** Filament tracer image of the seven voxel clusters in panel **(G)** in *en face* projection (Franklin and Paxinos, 2007).

To ask whether BRIAN could be effective when axons were more densely labeled and intermingled, we moved to a region in which most of the retinal input comes from ipRGCs, the suprachiasmatic nucleus (SCN). We imaged a 605 μm × 707 μm × 57 μm ROI encompassing both hemispheres of the SCN in an *Opn4*^Cre/+^*;rd/rd* mouse. We filtered the resulting image using the same approach as previously described (Figure 7A) and ran this region through our 3D PCA (Figure 7B). We were able to isolate seven voxel clusters with good confidence. Images corresponding to two of these clusters, and associated traced filaments are shown in Figures 7C–F. Voxels belonging to the red cluster were traced using the Filament tracer to reveal 3 long continuous filaments (which may represent the axonal projections of three cells with similar color or parts of the same neuron that would be continuous if viewed in a larger brain volume) Figure 7D. One of the three contiguous filaments had a total length of 4,047 μm and 46 branch points and was found to innervate both hemispheres (Supplementary Figure 4) in accordance with a previous report of ipRGC input to the SCN based upon sparse labeling (Fernandez et al., 2016). Using the same approach, all the seven identified clusters were extracted and recreated in 3D (Figure 7G) and then traced with Filament tracer to provide a detailed representation of axonal projections to this nucleus (Figure 7H and Supplementary Video 3).

## Discussion

During advanced retinal degeneration, the death of rod and cone photoreceptors leaves ipRGCs providing the only source of light information to the brain. In the healthy retina, these cells influence a wide variety of physiological and behavioral responses to light, and this is reflected in the distinct morphological differences between the six subtypes and their projection targets (Berson et al., 2010; Schmidt et al., 2011; Estevez et al., 2012; Stabio et al., 2018; Quattrochi et al., 2019). Thus, characterizing, and quantifying ipRGC morphology contributes to our understanding of the impact retinal degeneration may have on mammalian physiology and behavior. To answer this question, we developed a computational toolbox (BRIAN) that first pre-processes and cleans images, before segmenting them based upon identification of voxel clusters in principal component space and integrates with a commercial semi-automated tracing process to provide anatomically accurate reconstructions, efficiently, with single cell resolution, and in 3D. We apply BRIAN to produce 3D images of whole single ipRGCs with extensive and complex dendritic arborizations in the retina and show these reconstructed images retain sufficient resolution to allow quantitative morphological analysis of all six subtypes (M1−M6) and to establish their anatomical relationships in 3D. We apply BRIAN to produce 3D images of whole single ipRGCs with extensive and complex dendritic arborizations in the degenerate retina and show these reconstructed images retain sufficient resolution to allow identification and quantitative morphological analysis of all six subtypes (M1−M6) and to establish their anatomical relationships in 3D. We further show that BRIAN can be applied to reconstruct ipRGC projections in two brain nuclei, even when these are complex, extend over large volumes of tissue, and are densely intermingled.

### BRIAN: A computational platform for analyzing multi-color labeled tissue

Brainbow and the growing variety of related multi-reporter labeling techniques represent an exciting opportunity to describe neuronal ensembles with single cell resolution. There have been substantial technological advances in labeling methods (Garcia-Moreno et al., 2014; Loulier et al., 2014; Dumas et al., 2015; Viswanathan et al., 2015; Sakaguchi et al., 2018) and microscopy techniques (Lakadamyali et al., 2012; Chen et al., 2015; Abdeladim et al., 2019) for these approaches, but there remains a fundamental bottleneck in efficiently, reliably and objectively segmenting the resultant images. The technologically simplest approach to analyzing Brainbow labeled images is to rely on a human user to identify the color of an individual neuron by eye, and manually trace its extensions by linking image elements of similar color and continuity in space (Pan et al., 2013; Robles et al., 2013; Hammer et al., 2015; Herget et al., 2017). In addition to being subjective and non-documentable, that approach can be extremely labor intensive and rapidly becomes unfeasible when working in 3D, when labeled processes are strongly intermingled, or where neuronal architecture is complex and/or covers large brain volumes. Recent advances have concentrated on improving methods for simultaneous segmentation of the image based upon color and spatial location, by providing enhanced tools for a user to apply in identifying and tracing elements of a single neuron, or by attempting a method to fully automate that process (Sumbul et al., 2016; Roossien et al., 2019). The latter still presents the user with a challenging image annotation task, especially when working with neurons with a complex 3D architecture, while fully automated methods have not seen application and appear sensitive to color variation within a cell.

In developing BRIAN, we reasoned that it may be possible to sufficiently segment many Brainbow images based solely upon color whilst taking advantage of existing software packages that allow accurate and efficient neuronal tracing in more sparsely labeled samples. Our strategy therefore was to generate simplified versions of a ROI comprising voxel of similar “color” (and therefore corresponding to the elements of one or a few cells) that were suitable for tracing with a commercially available product (Filament Tracer). In searching for voxel clusters of similar “color” in any given image, BRIAN applies PCA to produce the most informative representation of variation in reporter fluorescence. Current analyses employ the ratio of reporter gene expression (as represented by “color”) to identify and link elements of a cell (Pan et al., 2013; Robles et al., 2013; Hammer et al., 2015; Sumbul et al., 2016; Wang et al., 2017). This strategy is according to the central premise of multi-color labeling approaches that, while absolute fluorescence intensity may vary across a cell, the relative expression of each fluorescent reporter is retained (Livet et al., 2007; Cai et al., 2013). However in reality, the ratio of reporter fluorescence (“color”) is quite variable across voxels from a single cell (Livet et al., 2007; Hammer et al., 2015; Sumbul et al., 2016) resulting in segmentation errors (Livet et al., 2007; Sumbul et al., 2016; Roossien et al., 2019). The PC space employed by BRIAN allows both color and brightness to be included in the color separation step and confirms that color inconsistency is to a substantial extent predictable. Voxel distribution plots in 3D PC space reveal that clusters exist as vectors in which ratio of fluorescence across the reporter protein channels varies systematically as a function of overall fluorescence intensity. This likely reflects non-linearities in labeling and/or image acquisition associated with threshold and saturation effects. The tapered polygon cluster identification and extraction tool in BRIAN accounts for this fundamental property of input images and returns a sufficient fraction of the voxels for individual cells to successfully recreate their morphology in output images.

In its current iteration BRIAN is integrated with the commercially available software package Filament Tracer (Imaris; Bitplane; Zurich) to create a seamless transition through the steps from image input to generation of a final composite image comprising multiple traced cells and associated quantitative morphological information. Using Filament tracer allowed us to conduct both automated and semi-automated 3D tracing on the simplified images. However, with appropriate modifications, versions of BRIAN which are integrated with other tracing options are entirely feasible given the modular nature of the process. We found the 3D nature of output images, where isolated voxel clusters are plotted in their original location in X, Y, and Z dimensions, to be critical in allowing user input to the tracing step, allowing the user to rotate the segmented image in all 3 dimensions to identify the best vantage point for tracing. This capacity is essential when reconstructing complex neuronal architecture with axons and/or dendritic processes moving in X, Y, and Z dimensions in unpredictable ways. The color-based segmentation of the starting image is critical in allowing such a 3D tracing strategy, as starting images were much too complex to trace reliably and objectively in this way. It offers additional functionality over alternatives in which the process of tracing is simplified by either collapsing the image to 2D (Robles et al., 2013) (thus losing 3D information) or by tracing in a series of 2D image stacks (Herget et al., 2017; Roossien et al., 2019), which is hard to achieve when fibers take complex routes through the ROI. The ability to trace in all 3D spatial dimensions simultaneously is also critical when handling axonal projections, in which fibers running principally along the z dimension could be very hard to detect in 2D hyper-stacks. Fully automated image segmentation is the ultimate goal, and this has been approached based upon applying spatio-color relations to link voxels from individual cells (Bas and Erdogmus, 2010; Shao et al., 2012; Sumbul et al., 2016; Duan et al., 2021). However, while such automated image segmentation is at the technology development, rather than application stage, and the success of current iterations is constrained by variations in color within single neurons, The current alternative to multi-color labeling approaches is monochromatic sparse labeling, which can resolve the anatomy of individual neurons, but only when processes are not overlapping to ensure accurate single cell reconstruction. BRIAN, importantly, accommodates intertwined processes which not only increases the throughput of single cell reconstructions, but also allows for the anatomical associations between neighboring cells to be resolved without the need for *a prior* knowledge of its morphology. BRIAN therefore represents a viable method to exploit BRAINBOW, and other multi-color labeling approaches which are based on stochastic expression of multiple reporter proteins (Snippert et al., 2010; Loulier et al., 2014; Weissman and Pan, 2015; Dumas et al., 2022), in order to obtain quantitative morphological information from neuronal populations.

### Quantitative morphological analysis of ipRGCs in the retina and brain of *Opn4^Cre/+^*;*rd/rd* mouse

Previous reports of ipRGCs in retinal degeneration have been limited to soma counting following antibody labeling (Semo et al., 2003; Vugler et al., 2008), without detailed descriptions of dendritic morphology, and have not encompassed the more recently discovered ipRGC subtypes (M3−M6) which are not readily identifiable by immunostaining (Ecker et al., 2010; Schmidt and Kofuji, 2011; Estevez et al., 2012; Quattrochi et al., 2019). Our success in aligning the morphological characteristics (soma size, dendritic field size, complexity, and stratification) of BRIAN-reconstructed ipRGCs in the *rd/rd* retina with published work in intact retinas (Berson et al., 2010; Ecker et al., 2010; Schmidt et al., 2011; Quattrochi et al., 2019) reveals that all main morphological classes are retained in advanced retinal degeneration. Thus, of 35 reconstructed ipRGCs from 4 *rd/rd* retinas, 34 could be assigned to one of the M1−M6 morphological sub-divisions of ipRGC, including examples of the rare bistratified M3 and M6 subtypes. For these cells, morphological characteristics including soma size, stratification, and complexity were similar to those reported in the visually intact animals for each defined subtype (Ecker et al., 2010; Schmidt and Kofuji, 2011; Estevez et al., 2012; Quattrochi et al., 2019), extending previous reports that the M1 & M2 subtype are not grossly affected by outer retinal degeneration (Vugler et al., 2008). We did note that, on average, the dendritic field size of our reconstructed ipRGCs was smaller for each subtype compared to published literature for visually intact mice (Table 1). Whilst this could be caused by differences in tissue preparation, as we used fixed tissue whereas previous reports are from live cell imaging (Ecker et al., 2010; Estevez et al., 2012; Quattrochi et al., 2019), it has been reported that up to 50% of all RGC subtypes possess an undersized dendritic field in retinal degeneration, which is likely a consequence of incomplete development due to the loss of visual input during the critical period of arbor refinement (Damiani et al., 2012). We did find one labeled ipRGC in the *rd/rd* retina that did not clearly fit into any of the six defined ipRGC subtypes in mouse described in the literature, thanks to its very large dendritic field size. Interestingly, a weakly expressing melanopsin cell type with similar characteristics has been reported in the Human retina (Hannibal et al., 2017). These so called “Gigantic M1” cells possess large oval somas (30 μm) with large and sparse dendritic fields (>600 um) that stratify exclusively in the OFF sublamina of the IPL (Hannibal et al., 2017), matching the characteristics of our mouse cell. Little is known about this recently identified subtype; however, this would be the first report of its existence in the mouse retina.

The six morphologically distinct ipRGC subtypes, and their subtype specific projection targets in the brain, are involved in a wide variety of physiological and behavioral responses to light and it is therefore of vital importance to understand how retinal degeneration affects this cell type. Previous studies have demonstrated that melanopsin-driven light responses in the degenerate retina show poor fidelity in response to repeated light exposure and slow temporal kinetics (Brown et al., 2010; Procyk et al., 2015; Eleftheriou et al., 2020), however our findings indicate this likely reflects the functional consequences of deafferentation, rather than morphological abnormalities.

It has been demonstrated that a single ipRGC projection can innervate multiple brain nuclei (Fernandez et al., 2016) and therefore the ultimate goal using further iterations of the BRIAN platform would be to characterize the morphology of individual ipRGC projections in retinorecipient nuclei, and pair them with specific subtypes as defined in the retina. Do different ipRGC subtypes exhibit different morphologies in the same nucleus? Does a single ipRGC innervate multiple nuclei and exhibit different morphologies for differing physiological and behavioral responses to light? How are they affected following retinal degeneration? These data would further our understanding of the importance of melanopsin signaling in health and disease, how light information is integrated within different retinorecipient nuclei and therefore more readily identify the role and function of individual ipRGCs with subtype specific resolution. It has recently been shown that ipRGCs possess synaptic specializations in brain targets using genetically encoded electron microscopy tag in ipRGCs and serial block-face electron microscopy (Kim et al., 2019). Combining our approach with synaptic markers in the future would allow further identification of the synaptic specializations of ipRGCs in different brain targets together with gross morphology mapping of their projections and therefore provide a deeper insight into structure-function relationship.

## Methods

### Animal housing

Mice were bred at the University of Manchester and housed under a 12:12 light/dark cycle, with food and water available *ad libitum*. *Opn4^Cre/+^*;*rd/rd* mice were created in house by crossing an established colony of *Opn4^Cre/+^* mice (kind gift of Dr. Samer Hattar, Johns Hopkins University) with commercially available C57 *rd*^1^ mice (Stock #: 000659; Jackson Laboratories). *Grm6^Cre/+^* mice (kind gift from Robert Duvoisin and R. Lane Brown, Oregon Health and Science University) on a mixed C57/Bl6 × C3H strain background were used for bipolar cell experiments. *Grm6^Cre/+^* mice used in this study are visually intact and do not possess the *Pde6b^rd^*^1^ mutation for retinal degeneration. All mice used in these experiments were greater than 4 months old at the time of injection to ensure outer photoreceptor degeneration was near completion and the inner retina was in a stable state of degeneration (Carterdawson et al., 1978; Jones and Marc, 2005). All procedures conformed to requirements of the United Kingdom Animals (Scientific Procedures) Act, 1986.

### Intravitreal injections

Two *Opn4^Cre/+^* mice and five *Opn4^Cre/+^*;*rd/rd* mice (>4 months old) mice were anesthetized with an intraperitoneal injection of Ketamine (100 mg/kg) and Xylazine (10 mg/kg). Once anesthetized, one drop of 1% Tropicamide and 2.5% Phenylephrine (Sigma Aldrich; United Kingdom) was topically applied to the left eye to fully dilate the pupil. A fine Hamilton RN needle 34 gauge fitted to a 5 μl Hamilton glass syringe was passed through the equator of the sclera (*Ora serrata*) and into the vitreous cavity whilst being careful to avoid the lens. 3 μl of solution comprising 1 μl each of floxed Brainbow virus (AAV9.hEF1a.lox.TagBFP.lox.eYFP.lox.WPRE.hGH-InvBYF and AV9.hEF1a.lox.mCherry.lox.mTFP1.lox.WPRE.hGH-InvCheTF; 10^13^ genomic particles/ml; Penn Vector Core, United States) and a 1 μl mixture of Heparinase III (200 units/ml; Sigma Aldrich, United Kingdom) and Hyaluronan lyase (200 units/ml; Sigma Aldrich, United Kingdom) (Cehajic-Kapetanovic et al., 2018) were introduced slowly over the course of 1 min to minimize reflux. Once complete, the needle was gently removed and a topical analgesic (0.25% Bupivacaine; Sigma Aldrich; United Kingdom) was applied to the injected eye. Mice were allowed to recover on a homeothermic heat mat before being returned to the colony room. One *Grm6^Cre/+^* mouse was anesthetized with an intraperitoneal injection of Ketamine (75 mg/kg) and Medetomidine (1 mg/kg) then recovered with 3 mg/kg Atipamezole. The mixture of Brainbow viruses was injected without enzymes with the total volume of ∼2.5 ul.

The 4−6 week after injection, mice were transcardially perfused with 0.1 M PBS followed by 4% methanol- free Paraformaldehyde (4% mf-PFA; Sigma Aldrich, United Kingdom). Immediately following perfusion, eyes and brains were removed and post-fixed in 4% methanol-free PFA overnight at 4°C. The retina from the injected eye was carefully dissected from the eye cup and stored in 4% PFA before undergoing immunohistochemistry as a free floating retinal wholemount. For anatomical tracing, post-fixed brains were cryoprotected in a 30% sucrose solution before sectioning (thickness 100 μm) on a cryostat (Microm HM560s) in either coronal or sagittal planes and stored in 0.1M PBS at 4°C until undergoing immunohistochemistry as free-floating sections in the order they were cut (rostral to caudal or lateral to medial).

### Immunocytochemistry

Both retinal wholemounts and brain sections underwent immunohistochemistry for the eYFP, mCherry, mTFP and tagBFP proteins. Tissue was initially permeabilized in a 1% TritonX-100 solution in PBS (1% PBS-X; PBS−Phosphate-buffered saline; 3 × 10 mins) before being blocked in a mixture of 5% donkey serum (Sigma Aldrich, United Kingdom) and 5% goat serum (Sigma Aldrich, United Kingdom) in 0.2% PBS-X for 3 h at room temperature. Tissue was subsequently incubated with primary antibodies against eYFP (chicken anti-GFP; 1:500; Kerafast), mCherry (rabbit anti-mCherry; 1:500; Kerafast), tagBFP (guinea pig anti-TFP; 1:500; Kerafast) and mTFP (rat anti-TFP; 1:500; Kerafast) for 3 days at 4°C. After this time, sections were washed thoroughly in 0.2% PBS-X before being incubated in their respective secondary antibodies: eYFP (Alexa-488 conjugated goat anti-Chicken; 1:200; Life technologies), mCherry (Alexa- 546 conjugated donkey anti-rabbit; 1:200; Life Technologies), mTFP (Alexa-647 conjugated donkey anti-rat; 1:200; Life technologies) and tagBFP (Alexa-594 conjugated goat anti-guinea pig; 1:200; Life Technologies) for 12 h at 4°C. Sections were then thoroughly washed in 0.2% PBS-X before undergoing one final wash in dH20. Wholemount retinas were placed onto glass slides with the ganglion cell layer facing up. The retina was cut into a Maltese cross motif before being mounted with Prolong Gold (Invitrogen, United Kingdom), coverslipped and left to dry. Brain sections were mounted sequentially from the order they were cut (lateral to medial or rostral to caudal) and mounted in the same orientation throughout. Once dry, sections were mounted with Prolong Gold (Invitrogen, United Kingdom), coverslipped and left to dry overnight in the dark at room temperature. Important to note, we found that the signal for tagBFP was much lower than for the other 3 proteins (note high background signal in Figure 1B). As a result, the tagBFP channel was less informative in isolating single cells in this study and that is why it was excluded from the rest of the study.

### Confocal laser scanning microscopy

3D images were acquired using a Leica TCS SP5 AOBS and Leica TCS SP8 AOBS inverted confocal microscopes equipped with a x63 x/0.50 Plan Fluotar objective. The confocal settings were as follows: pinhole 1 airy unit, scan speed 1000 Hz bidirectional, format 1024 × 1024. Images for each reporter protein were collected in individual channels using the following detection band settings; 493−520 nm; 569−588 nm; 598−620 nm; and 643−750 nm whilst utilizing the 488 nm and 564 nm, 594 nm and 637 nm laser lines, respectively. The power of individual laser lines was adjusted for each experiment to maximize the intensity range in each recorded channel whilst minimizing saturation. All images were acquired as 16-bit monochrome images via photomultiplier tube (PMT) detectors and pseudocolored during image acquisition. To eliminate crosstalk between color channels, images were collected sequentially. For acquiring 3D optical stacks, Z-depth was maintained at 1 μm for both retinas and brain sections. To gain high magnification images over a wide area of target tissue, we utilized the motorized stage and tile-scanning function in the confocal software (Leica AS). Individual tiles were imaged x63 magnification through the Z-stack and subsequently stitched back together in X, Y, and Z dimensions following image acquisition to represent a large region of the target tissue with high spatial resolution (voxel dimensions = 0.24 × 0.24 × 1.0 μm in X, Y, and Z).

### Image analysis

Our approach to analyzing Brainbow images can be divided into three major operations: (i) Pre-filtering of the acquired image; (ii) identification of voxel clusters by principal component analysis (PCA); and (iii) post-filtering and plotting of the clustered voxels in 3D space. Analysis code is available at: https://github.com/lucasgroup/BRIAN.

#### BRIAN: Pre-filtering and image preparation

The first aim of pre-filtering was to reduce background noise. Raw (.lif) files of retinal wholemounts and LGN tile scans were opened in Imaris 8.3 (Bitplane, Zurich). ROIs were identified as regions of a wholemount retina with a high concentration of stochastically labeled cells and were cropped in X, Y and (in the case of retinal images) Z dimensions to encompass labeled cells and/or their projections. All voxels which encompassed the ROI for each of the reporter proteins were sent to Python for processing using the ImarisXT module, and analyzed using a custom written function in C and OpenMP (Open Multi-processing) and interfaced with Python via the NumPy library (van der Walt et al., 2011). This function filtered noise in the ROI using several different parameters. Firstly, we applied an undecimated multi- dimension wavelet transform (“à trous” wavelet based on the separable linear 3 × 3 × 3 kernel and compensated for Z anisotropy) to each of the imaged channels, both to remove high frequency noise from the image, and to remove dim voxels from our image which corresponded to background noise and auto-fluorescence from the tissue. This was achieved by setting a threshold as a percentage of the full intensity scale (0−65,535 in a 16-bit image) in each of the resulting channels. An optional additional simplification of the images prior to cluster identification by excluding voxels with signal from only one fluorophore was applied in some analyses. Processed images were finally sent back to Imaris for 3D reconstruction to allow visual confirmation that adequate thresholding of the image had been achieved.

#### BRIAN: PCA analysis and cluster identification

The PCA transformation is a statistical technique that creates, from a linear combination of input variables, a new set of orthogonal dimensions (“Principal Components”) that are ranked by decreasing variance. The data is, in effect, rescaled, rotated and reflected such that it provides the most informative representation of the variation in the data set. The input variables for the PCA analysis consist of the intensity values of the reporter protein fluorescence recorded in each individual channel of the confocal microscope from our pre-filtered ROI. Voxels from our filtered 3D ROI were imported to Python, where the NumPy library was used to perform a principal component analysis on all voxels of the filtered image which had a strictly positive value. These new voxel values were plotted in principal component space and viewed as either a 3-Dimensional plot (PC1 vs. PC2 vs. PC3) or 3 interactive 2D plots (PC1 vs. PC2, PC1 vs. PC3 and PC2 vs. PC3) using the Matplotlib Python library (Hunter, 2007). To allow the programme to be interactive, we plotted a maximum of 80,000 pixels in each of our 2D comparisons of principal component space as we found this was suitable for reliable extraction, whilst maintaining a practical refresh rate for graph generation and live updates of the 2D projection. 3D representations of principal component space had the value for all voxels in the image plotted.

In the Brainbow strategy, voxels from a single cell are expected to share a common ratio of reporter gene expression but could vary in absolute brightness. In practice, the variation in brightness can impact the fluorescence signal ratio because of non-linearities in the relationship between the amount of reporter protein and the magnitude of recorded fluorescence caused by threshold and saturation effects in image capture (simulated in Supplementary Figures 1A–H). In this way, the range of potential ratios of reporter gene fluorescence becomes constrained as brightness becomes either too high or low. At the extremes voxels from all cells converge on the black and white points for the image. This theoretical behavior was indeed observed in our data, as we found that when plotted in PC space, voxels from a single neuron could fall upon a vector projecting from the calculated black point (the location of voxels with intensity value of 0 for each channel) and/or another vector converging on the calculated white point (location of voxels with maximum intensity in all channels). Keeping this behavior in mind, we generated a graphical user interface (GUI) to interactively select voxels within our 2D principal component plots. The GUI included a template for capturing clusters with user- defined parameters. In designing a shape to use as an extraction template we were mindful: (1) that voxels became more densely clustered as the black point was approached; and (2) that in images with higher brightness, voxels within this cluster should also approach the white point (Supplementary Figures 1D,E). For these reasons, we employed an extraction template comprising a tapered polygon projecting from black and white points meeting at a user defined “elbow” point somewhere between the two. The location of the “elbow” point and width of the polygons were defined by the user to maximize the fraction of voxels within the cluster captured, whilst minimizing contamination from other voxels. The basic shape of the template was a 2-dimensional polygon built around a pair of lines extending from the black point (Supplementary Figure 1F) and white points (Supplementary Figure 1G) and meeting in the middle. The user was able to define the point at which the two lines met (handle point), which in turn determined their vector directions. The goal was to place this handle point such that the two component lines approximated the location of voxel clusters in PC space (Supplementary Figure 1H).

To ensure that a reasonable fraction of voxels comprising the cluster were captured, a polygon was built around this three-point polyline to define an area of PC space whose width was greatest at the handle point. The user could determine the width of the polygon at the handle point and the extent to which the polygon extended to black and white points by controlling interactive scales arbitrarily ranging from 0 to 200. This allowed for real-time adjustments to the dimensions of the polygon in principal component space. To define the location of voxels from a single cell in the 3D PC space, this polygon fitting had to be undertaken on at least two of the three 2D PC space representations. The goal was to do so in such a way as to ensure that voxels falling within both polygons came from a single neuron.

Two cues were available to help the user identify polygons that captured voxel clusters in the 2D PC spaces that corresponded to elements of a single cell. Firstly, we pseudocolored the voxels in the PC space to represent the relative fluorescent signal for the 3 reporter proteins by transforming into RGB and provided a bar which updated to show the “color” of voxels at the user defined handle point. In essence, this provided an additional dimension of separation for the voxels that was visible across each 2D plot. Secondly, we provided a live 2D maximum projection of the ROI displaying voxels falling within the user-defined polygons. All parameters for the PCA analysis and polyline co-ordinates were saved as html files for each ROI. A snapshot of the GUI features is shown in Supplementary Figure 2G.

#### BRIAN: Spatial reconstruction of single cells

Following isolation of an individual cluster the spatially indexed voxels were sent back to Imaris (version 8.3 or later) for 3D reconstruction and further processing. During this transfer, we once more used an undecimated wavelet transform to smooth over voxels which may not have come through the cluster identification process due to non-uniform expression of reporter protein across the length of the cell. For this task, we found that the Cohen-Daubechies-Feauveau (CDF) 9/7 kernel performed best by preserving fine structures of dendrites and interpolating across any missing voxels. Spatially reconstructed voxels which corresponded to an isolated cluster were then sent to Imaris as a new channel whose color was assigned the average RGB voxel value at the handle point in principal component space. We used the commercially available plug-in filament tracer (Imaris, Bitplane, Zurich) to trace the morphology of voxels representing a single cell in our new channel. Using the creation wizard, we utilized the automatic (no-loop) detection parameters to automatically trace the 3-Dimensional structure of our voxels isolated from the analysis. Contrast settings and seeding values were set independently for individual isolated cells in the filament tracer plug-in. We found that depending on the labeling and/or signal:noise the automated Filament tracer reconstruction could be erroneous when connecting fine structures of the isolated cell. Therefore, following this automated creation, the traced cell was manually inspected to ensure correct tracing and branch points were created by overlaying both the 3D isolated cell, and the original filtered RGB image. We used the “remove disconnected segments” function in addition to the “disconnect segments” and “join segments” function was used to improve the quality of the automated method and account for any discrepancies between the isolated cell and its automated reconstruction.

#### Quantitative morphological analysis of isolated cells

The dendrite beginning point was defined as the center of the soma when overlaid onto the reconstructed cell. For visualization purposes, the dendrite thickness was set to 3 μm and therefore does not reflect the true thickness of individual cells. Images of the spatially reconstructed pixels from a cluster and their corresponding filament tracer were represented as 2 dimensional snapshots in both the X, Y and X, Z dimensions in Imaris. Quantitative statistics for dendritic length, branch points and Sholl analysis were calculated in Filament Tracer and statistical values were exported and stored in Microsoft Excel 2007. Soma size and dendritic field size was calculated from the 2D projection of the isolated channel in ImageJ (Schindelin et al., 2012) and confirmed on the 2D projection of the reconstructed cell. For an individually isolated cell, we traced the outline using a minimal convex polygon enclosing on the cell body or the tips of the dendrites. We measured the area of this polygon in ImageJ and calculated the diameter of a circle of equal area as previously described (Berson et al., 2010). All graphs were generated in Graphpad Prism 7.

## Data availability statement

The original contributions presented in this study are included in the article/Supplementary material, further inquiries can be directed to the corresponding author.

## Ethics statement

This animal study was reviewed and approved by the Home Office, United Kingdom, PP3176367.

## Author contributions

CP, EZ, RL, and NM did the conception and design of the work. CP, NM, JR, and EZ did the acquisition, analysis, and interpretation of the data. CP, EZ, RL, and NM contributed to the drafting and revising work critically for important intellectual content. All experiments were carried out at the University of Manchester in the laboratory of RL. All authors approved the final version of the manuscript.

## References

[B1] Abdeladim L. Matho K. S. Clavreul S. Mahou P. Sintes J. M. Solinas X. (2019). Multicolor multiscale brain imaging with chromatic multiphoton serial microscopy. *Nat. Commun.* 10:2160. 10.1038/s41467-019-09552-9 31073140 PMC6509334

[B2] Allen A. E. Martial F. P. Lucas R. J. (2019). Form vision from melanopsin in humans. *Nat. Commun.* 10:2274. 10.1038/s41467-019-10113-3 31118424 PMC6531428

[B3] Allen A. E. Storchi R. Martial F. P. Bedford R. A. Lucas R. J. (2017). Melanopsin contributions to the representation of images in the early visual system. *Curr. Biol.* 27 1623.e–1632.e. 10.1016/j.cub.2017.04.046 28528909 PMC5462620

[B4] Bas E. Erdogmus D. (2010). “Piecewise linear cylinder models for 3-dimensional axon segmentation in brainbow imagery,” in *Proceeding of the 2010 7th IEEE international symposium on biomedical imaging: From Nano to Macro*, (Rotterdam: IEEE), 1297–1300. 10.1109/ISBI.2010.5490234

[B5] Berson D. M. Castrucci A. M. Provencio I. (2010). Morphology and mosaics of melanopsin-expressing retinal ganglion cell types in mice. *J. Comp. Neurol.* 518 2405–2422. 10.1002/cne.22417 20503419 PMC2895505

[B6] Berson D. M. Dunn F. A. Takao M. (2002). Phototransduction by retinal ganglion cells that set the circadian clock. *Science* 295 1070–1073. 10.1126/science.1067262 11834835

[B7] Brown T. M. Gias C. Hatori M. Keding S. R. Semo M. Coffey P. J. (2010). Melanopsin contributions to irradiance coding in the thalamo-cortical visual system. *PLoS Biol.* 8:e1000558. 10.1371/journal.pbio.1000558 21151887 PMC2998442

[B8] Cai D. Cohen K. B. Luo T. Lichtman J. W. Sanes J. R. (2013). Improved tools for the brainbow toolbox. *Nat. Methods* 10 540–547. 10.1038/nmeth.2450 23817127 PMC3713494

[B9] Carterdawson L. D. Lavail M. M. Sidman R. L. (1978). Differential effect of rd mutation on rods and cones in mouse retina. *Invest. Ophthalmol. Vis. Sci.* 17 489–498. 659071

[B10] Cehajic-Kapetanovic J. Milosavljevic N. Bedford R. A. Lucas R. J. Bishop P. N. (2018). Efficacy and safety of glycosidic enzymes for improved gene delivery to the retina following intravitreal injection in mice. *Mol. Ther. Methods Clin. Dev.* 9 192–202. 10.1016/j.omtm.2017.12.002 29766027 PMC5948313

[B11] Chang B. Hawes N. L. Hurd R. E. Davisson M. T. Nusinowitz S. Heckenlively J. R. (2002). Retinal degeneration mutants in the mouse. *Vis. Res.* 42 517–525. 10.1016/S0042-6989(01)00146-8 11853768

[B12] Chen F. Tillberg P. W. Boyden E. S. (2015). Expansion microscopy. *Science* 347 543–548. 10.1126/science.1260088 25592419 PMC4312537

[B13] Cui Q. Ren C. Sollars P. J. Pickard G. E. So K. F. (2015). The injury resistant ability of melanopsin-expressing intrinsically photosensitive retinal ganglion cells. *Neuroscience* 284 845–853. 10.1016/j.neuroscience.2014.11.002 25446359 PMC4637166

[B14] Damiani D. Novelli E. Mazzoni F. Strettoi E. (2012). Undersized dendritic arborizations in retinal ganglion cells of the rd1 mutant mouse: A paradigm of early onset photoreceptor degeneration. *J. Comp. Neurol.* 520 1406–1423. 10.1002/cne.22802 22102216 PMC4112223

[B15] Duan B. Walker L. A. Roossien D. H. Shen F. Y. Cai D. W. Yan Y. (2021). “Unsupervised neural tracing in densely labeled multispectral brainbow images,” in *Proceeding of the 2021 IEEE 18th international symposium on biomedical imaging (Isbi)*, (Nice: IEEE), 1122–1126. 10.1109/ISBI48211.2021.9433932

[B16] Dumas L. Clavreul S. Michon F. Loulier K. (2022). Multicolor strategies for investigating clonal expansion and tissue plasticity. *Cell. Mol. Life Sci.* 79:141. 10.1007/s00018-021-04077-1 35187598 PMC8858928

[B17] Dumas L. Heitz-Marchaland C. Fouquet S. Suter U. Livet J. Moreau-Fauvarque C. (2015). Multicolor analysis of oligodendrocyte morphology. Interactions, and development with brainbow. *Glia* 63 699–717. 10.1002/glia.22779 25530205

[B18] Ecker J. L. Dumitrescu O. N. Wong K. Y. Alam N. M. Chen S. K. Legates T. (2010). Melanopsin-expressing retinal ganglion-cell photoreceptors: Cellular diversity and role in pattern vision. *Neuron* 67 49–60. 10.1016/j.neuron.2010.05.023 20624591 PMC2904318

[B19] Eleftheriou C. G. Wright P. Allen A. E. Elijah D. Martial F. P. Lucas R. J. (2020). Melanopsin driven light responses across a large fraction of retinal ganglion cells in a dystrophic retina. *Front. Neurosci.* 14:320. 10.3389/fnins.2020.00320 32317928 PMC7147324

[B20] Estevez M. E. Fogerson P. M. Ilardi M. C. Borghuis B. G. Chan E. Weng S. J. (2012). Form and function of the m4 cell, an intrinsically photosensitive retinal ganglion cell type contributing to geniculocortical vision. *J. Neurosci.* 32 13608–13620. 10.1523/JNEUROSCI.1422-12.2012 23015450 PMC3474539

[B21] Fernandez D. C. Chang Y. T. Hattar S. Chen S. K. (2016). Architecture of retinal projections to the central circadian pacemaker. *Proc. Natl. Acad. Sci. U.S.A.* 113 6047–6052. 10.1073/pnas.1523629113 27162356 PMC4889372

[B22] Franklin K. B. J. Paxinos G. (2007). *The mouse brain: Brain Atlas*.

[B23] Freedman M. S. Lucas R. J. Soni B. Von Schantz M. Munoz M. David-Gray Z. (1999). Regulation of mammalian circadian behavior by non-rod, non-cone, ocular photoreceptors. *Science* 284 502–504. 10.1126/science.284.5413.502 10205061

[B24] Garcia-Moreno F. Vasistha N. A. Begbie J. Molnar Z. (2014). CloNe is a new method to target single progenitors and study their progeny in mouse and chick. *Development* 141 1589–1598. 10.1242/dev.105254 24644261 PMC3957378

[B25] Hammer S. Monavarfeshani A. Lemon T. Su J. M. Fox M. A. (2015). Multiple retinal axons converge onto relay cells in the adult mouse thalamus. *Cell Rep.* 12 1575–1583. 10.1016/j.celrep.2015.08.003 26321636 PMC5757867

[B26] Hampel S. Chung P. Mckellar C. E. Hall D. Looger L. L. Simpson J. H. (2011). Drosophila brainbow: A recombinase-based fluorescence labeling technique to subdivide neural expression patterns. *Nat. Methods* 8 253–U102. 10.1038/nmeth.1566 21297621 PMC3077945

[B27] Hannibal J. Christiansen A. T. Heegaard S. Fahrenkrug J. Kiilgaard J. F. (2017). Melanopsin expressing human retinal ganglion cells: Subtypes, distribution, and intraretinal connectivity. *J. Comp. Neurol.* 525 1934–1961. 10.1002/cne.24181 28160289

[B28] Hattar S. Kumar M. Park A. Tong P. Tung J. Yau K. W. (2006). Central projections of melanopsin-expressing retinal ganglion cells in the mouse. *J. Comp. Neurol.* 497 326–349. 10.1002/cne.20970 16736474 PMC2885916

[B29] Hattar S. Liao H. W. Takao M. Berson D. M. Yau K. W. (2002). Melanopsin-containing retinal. ganglion cells: Architecture, projections, and intrinsic photosensitivity. *Science* 295 1065–1070. 10.1126/science.1069609 11834834 PMC2885915

[B30] Herget U. Gutierrez-Triana J. A. Thula O. S. Knerr B. Ryu S. (2017). Single-cell reconstruction of oxytocinergic neurons reveals separate hypophysiotropic and encephalotropic subtypes in larval zebrafish. *Eneuro* 4:ENEURO.278–ENEURO.216. 10.1523/ENEURO.0278-16.2016 28317020 PMC5356222

[B31] Hunter J. D. (2007). Matplotlib: A 2D graphics environment. *Comp. Sci. Eng.* 9 90–95. 10.1109/MCSE.2007.55

[B32] Jones B. W. Marc R. E. (2005). Retinal remodeling during retinal degeneration. *Exp. Eye Res.* 81 123–137. 10.1016/j.exer.2005.03.006 15916760

[B33] Kim K. Y. Rios L. C. Le H. Perez A. J. Phan S. Bushong E. A. (2019). Synaptic specializations of melanopsin-retinal ganglion cells in multiple brain regions revealed by genetic label for light and electron microscopy. *Cell Rep.* 29 628.e–644.e. 10.1016/j.celrep.2019.09.006 31618632 PMC7045601

[B34] Lakadamyali M. Babcock H. Bates M. Zhuang X. W. Lichtman J. (2012). 3D multicolor super-resolution imaging offers improved accuracy in neuron tracing. *PLoS One* 7:e30826. 10.1371/journal.pone.0030826 22292051 PMC3265519

[B35] Lin B. Peng E. B. (2013). Retinal ganglion cells are resistant to photoreceptor loss in retinal degeneration. *PLoS One* 8:e68084. 10.1371/journal.pone.0068084 23840814 PMC3695938

[B36] Livet J. Weissman T. A. Kang H. N. Draft R. W. Lu J. Bennis R. A. (2007). Transgenic strategies for combinatorial expression of fluorescent proteins in the nervous system. *Nature* 450 56–62. 10.1038/nature06293 17972876

[B37] Loulier K. Barry R. Mahou P. Le Franc Y. Supatto W. Matho K. S. (2014). Multiplex cell and lineage tracking with combinatorial labels. *Neuron* 81 505–520. 10.1016/j.neuron.2013.12.016 24507188

[B38] Lucas R. J. Hattar S. Takao M. Berson D. M. Foster R. G. Yau K. W. (2003). Diminished pupillary light reflex at high irradiances in melanopsin-knockout mice. *Science* 299 245–247. 10.1126/science.1077293 12522249

[B39] Morgans C. W. Zhang J. M. Jeffrey B. G. Nelson S. M. Burke N. S. Duvoisin R. M. (2009). Trpm1 is required for the depolarizing light response in retinal On-bipolar cells. *Proc. Natl. Acad. Sci. U.S.A.* 106 19174–19178. 10.1073/pnas.0908711106 19861548 PMC2776419

[B40] Nern A. Pfeiffer B. D. Rubin G. M. (2015). Optimized tools for multicolor stochastic labeling reveal diverse stereotyped cell arrangements in the fly visual system. *Proc. Natl. Acad. Sci. U.S.A.* 112 E2967–E2976. 10.1073/pnas.1506763112 25964354 PMC4460454

[B41] Pan Y. A. Freundlich T. Weissman T. A. Schoppik D. Wang X. C. Zimmerman S. (2013). Zebrabow: Multispectral cell labeling for cell tracing and lineage analysis in zebrafish. *Development* 140 2835–2846. 10.1242/dev.094631 23757414 PMC3678346

[B42] Pittler S. J. Baehr W. (1991). Identification of a nonsense mutation in the rod photoreceptor cgmp phosphodiesterase beta-subunit gene of the rd mouse. *Proc. Natl. Acad. Sci. U S.A.* 88 8322–8326. 10.1073/pnas.88.19.8322 1656438 PMC52500

[B43] Procyk C. A. Eleftheriou C. G. Storchi R. Allen A. E. Milosavljevic N. Brown T. M. (2015). Spatial receptive fields in the retina and dorsal lateral geniculate nucleus of mice lacking rods and cones. *J. Neurophysiol.* 114 1321–1330. 10.1152/jn.00368.2015 26084909 PMC4725120

[B44] Provencio I. Rodriguez I. R. Jiang G. S. Hayes W. P. Moreira E. F. Rollag M. D. (2000). A novel human opsin in the inner retina. *J. Neurosci.* 20 600–605. 10.1523/JNEUROSCI.20-02-00600.2000 10632589 PMC6772411

[B45] Quattrochi L. E. Stabio M. E. Kim I. Ilardi M. C. Fogerson P. M. Leyrer M. L. (2019). The M6 cell: A small-field bistratified photosensitive retinal ganglion cell. *J. Comp. Neurol.* 527 297–311. 10.1002/cne.24556 30311650 PMC6594700

[B46] Robles E. Filosa A. Baier H. (2013). Precise lamination of retinal axons generates multiple parallel input pathways in the tectum. *J. Neurosci.* 33 5027–5039. 10.1523/JNEUROSCI.4990-12.2013 23486973 PMC3641828

[B47] Roossien D. H. Sadis B. V. Van Y. Webb J. M. Min L. Y. Dizaji A. S. (2019). Multispectral tracing in densely labeled mouse brain with nTracer. *Bioinformatics* 35 3544–3546. 10.1093/bioinformatics/btz084 30715234 PMC6748755

[B48] Ruggiero L. Allen C. N. Brown R. L. Robinson D. W. (2010). Mice with early retinal degeneration show differences in neuropeptide expression in the suprachiasmatic nucleus. *Behav. Brain Funct.* 6:36. 10.1186/1744-9081-6-36 20604961 PMC2912232

[B49] Sakaguchi R. Leiwe M. N. Imai T. (2018). Bright multicolor labeling of neuronal circuits with fluorescent proteins and chemical tags. *Elife* 7:e40350. 10.7554/eLife.40350 30454553 PMC6245733

[B50] Schindelin J. Arganda-Carreras I. Frise E. Kaynig V. Longair M. Pietzsch T. (2012). Fiji: An open-source platform for biological-image analysis. *Nat. Methods* 9 676–682. 10.1038/nmeth.2019 22743772 PMC3855844

[B51] Schmidt T. M. Alam N. M. Chen S. Kofuji P. Li W. Prusky G. T. (2014). A role for melanopsin in alpha retinal ganglion cells and contrast detection. *Neuron* 82 781–788. 10.1016/j.neuron.2014.03.022 24853938 PMC4083763

[B52] Schmidt T. M. Kofuji P. (2009). Functional and morphological differences among intrinsically photosensitive retinal ganglion cells. *J. Neurosci.* 29 476–482. 10.1523/JNEUROSCI.4117-08.2009 19144848 PMC2752349

[B53] Schmidt T. M. Kofuji P. (2011). Structure and function of bistratified intrinsically photosensitive retinal ganglion cells in the mouse. *J. Comp. Neurol.* 519 1492–1504. 10.1002/cne.22579 21452206 PMC3714856

[B54] Schmidt T. M. Chen S. K. Hattar S. (2011). Intrinsically photosensitive retinal ganglion cells: Many subtypes, diverse functions. *Trends Neurosci.* 34 572–580. 10.1016/j.tins.2011.07.001 21816493 PMC3200463

[B55] Semo M. Peirson S. Lupi D. Lucas R. J. Jeffery G. Foster R. G. (2003). Melanopsin retinal ganglion cells and the maintenance of circadian and pupillary responses to light in aged rodless/coneless (rd/rdcl) mice. *Eur. J. Neurosci.* 17 1793–1801. 10.1046/j.1460-9568.2003.02616.x 12752778

[B56] Shao H. C. Cheng W. Y. Chen Y. C. Hwang W. L. (2012). “Colored multi-neuron image processing for segmenting and tracing neural circuits,” in *proceeding of the 19th IEEE international conference on image processing (Icip)*, (Lake Buena Vista, Fl: IEEE), 2025–2028.

[B57] Snippert H. J. Van Der Flier L. G. Sato T. Van Es J. H. Van Den Born M. Kroon-Veenboer C. (2010). Intestinal crypt homeostasis results from neutral competition between symmetrically dividing lgr5 stem cells. *Cell* 143 134–144. 10.1016/j.cell.2010.09.016 20887898

[B58] Stabio M. E. Sabbah S. Quattrochi L. E. Ilardi M. C. Fogerson P. M. Leyrer M. L. (2018). The M5 Cell: A color-opponent intrinsically photosensitive retinal ganglion cell. *Neuron* 97 150–163e4. 10.1016/j.neuron.2017.11.030 29249284 PMC5757626

[B59] Storchi R. Milosavljevic N. Eleftheriou C. G. Martial F. P. Orlowska-Feuer P. Bedford R. A. (2015). Melanopsin-driven increases in maintained activity enhance thalamic visual response reliability across a simulated dawn. *Proc. Natl. Acad. Sci. U.S.A.* 112 E5734–E5743. 10.1073/pnas.1505274112 26438865 PMC4620906

[B60] Sumbul U. Roossien D. Chen F. Barry N. Boyden E. S. Cai D. W. (2016). “Automated scalable segmentation of neurons from multispectral images,” in *Advances in neural information processing systems 29: Annual conference on neural information processing systems 2016*, eds Lee D. D. Sugiyama M. Luxburg U. V. Guyon I. Garnett R. (Barcelona), 1912–1920.

[B61] Tran N. M. Shekhar K. Whitney I. E. Jacobi A. Benhar I. Hong G. S. (2019). Single-cell profiles of retinal ganglion cells differing in resilience to injury reveal neuroprotective genes. *Neuron* 104 1039.e–1055.e. 10.1016/j.neuron.2019.11.006 31784286 PMC6923571

[B62] Tsukamoto Y. Omi N. (2017). Classification of mouse retinal bipolar cells: Type-specific connectivity with special reference to rod-driven aii amacrine pathways. *Front. Neuroanat.* 11:92. 10.3389/fnana.2017.00092 29114208 PMC5660706

[B63] van der Walt S. Colbert S. C. Varoquaux G. (2011). The NumPy Array: A structure for efficient numerical computation. *Comp. Sci. Eng.* 13 22–30. 10.1109/MCSE.2011.37

[B64] Viswanathan S. Williams M. E. Bloss E. B. Stasevich T. J. Speer C. M. Nern A. (2015). High-performance probes for light and electron microscopy. *Nat. Methods* 12 568–576. 10.1038/nmeth.3365 25915120 PMC4573404

[B65] Vugler A. A. Semo M. Joseph A. Jeffery G. (2008). Survival and remodeling of melanopsin cells during retinal dystrophy. *Vis. Neurosci.* 25 125–138. 10.1017/S0952523808080309 18442436

[B66] Wang J. J. O’sullivan M. L. Mukherjee D. Punal V. M. Farsiu S. Kay J. N. (2017). Anatomy and spatial organization of Muller glia in mouse retina. *J. Comp. Neurol.* 525 1759–1777. 10.1002/cne.24153 27997986 PMC5542564

[B67] Weissman T. A. Pan Y. A. (2015). Brainbow: New resources and emerging biological applications for multicolor genetic labeling and analysis. *Genetics* 199 293–306. 10.1534/genetics.114.172510 25657347 PMC4317644

