# Supplementary material for: Quantitative characterisation of ipRGCs in retinal degeneration using a computation platform for extracting and reconstructing single neurons in 3D from a multi-colour labeled population
Source: Front Cell Neurosci. 2022 Nov 1;16:1009321. doi: 10.3389/fncel.2022.1009321 (PMC9664085; doi:10.3389/fncel.2022.1009321)
Supplement: Supplementary file 1 [file Data_Sheet_1.pdf]

Supplementary Figures:

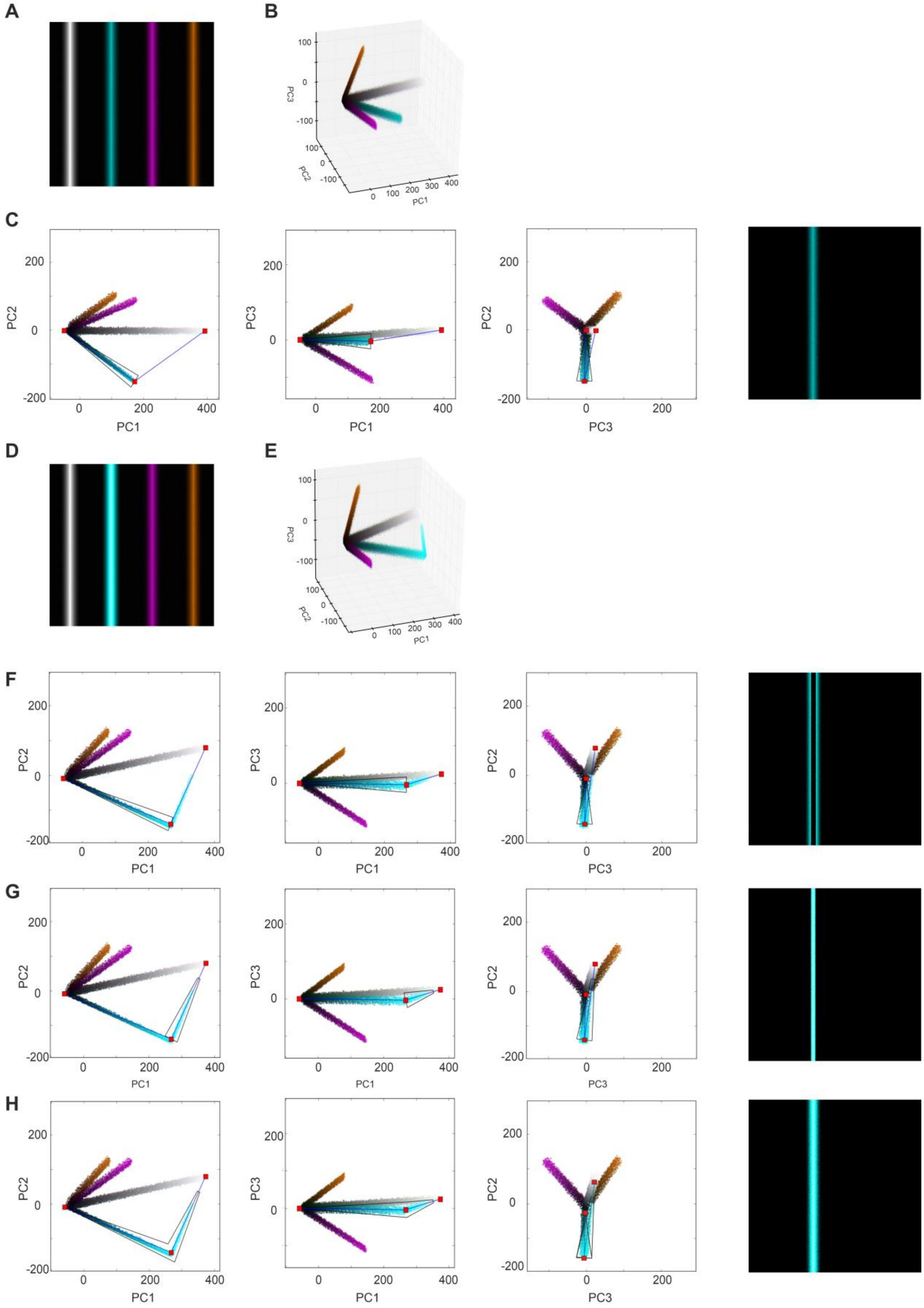

Supplementary Figure 1: Defining cluster isolation in PC Space using simulated images (A) *Computer generated image of four coloured bars (whose brightness increases from the outer edge to the centre) on a black background: Grey (0.5, 0.5, 0.5, 1.0), Cyan (0.0, 1.0, 1.0, 0.6), Purple (0.5, 0.0, 0.5, 0.6) and Orange (1.0, 0.5, 0.0, 0.6) (values represent 'RGBmax' quadruplet which is converted to a Hue, Saturation, Lightness (HSL) values. H and S were maintained but the value for L was varied between 0 (for the outer edges of the bar) to 1 (at the centre of the bar) to create a gradient so that the edges of the bar where black (no signal) and the centre was white (saturation)).* ***(B)*** *PCA conducted on the intensity values of the constituent pixels were plotted in a 3D representation of PC space (PC1 vs. PC2 vs. PC3) showing four clear clusters of voxels diverging from the 'black point' (0,0,0).* ***(C)*** *2D representations of the PC space (from left to right; PC1 vs. PC2, PC1 vs. PC3 and PC2 vs. PC3). The Cyan cluster can be clearly visualised in 2 of the 3 representations of PC space (PC1 vs. PC2 & PC2 vs. PC3). The 'handle point' was placed at the furthest point away from the black point and encompassed the cyan cluster of pixels. Spatial reconstruction of these pixels demonstrated that the cyan cluster represented the cyan bar in the original image.* ***(D)*** *The same computer-generated image as in (A) except the cyan bar is brighter and reaches a point of near saturation: Cyan (0.0, 1.00, 1.00, 0.90).* ***(E)*** *3D representation of PC space from the image in (D) shows the cyan cluster of voxels diverging from the black point, reaches a point of inflection, and tends towards the white point. This point of inflection is not present in (B).* ***(F)*** *We employed our extraction polygon in 3 x 2D PC space to isolate the cyan cluster up to the point of inflection and the resultant spatial reconstruction of these pixels demonstrated only the outer edges (dimmer regions) of the cyan bar.* ***(G)*** *We next extracted the cyan cluster from the point of inflection towards the white point and the resultant spatial reconstruction of these pixels demonstrated only the inner region (brighter region) of the cyan bar.* ***(H)*** *Extraction of the entire cyan cluster (from black point to the white point via the point in flection) is required to spatially reconstruct the entirety of the cyan bar. Analysis code for the interactive tool to explore clusters in these simulated images is available at* https://github.com/lucasgroup/BRIAN

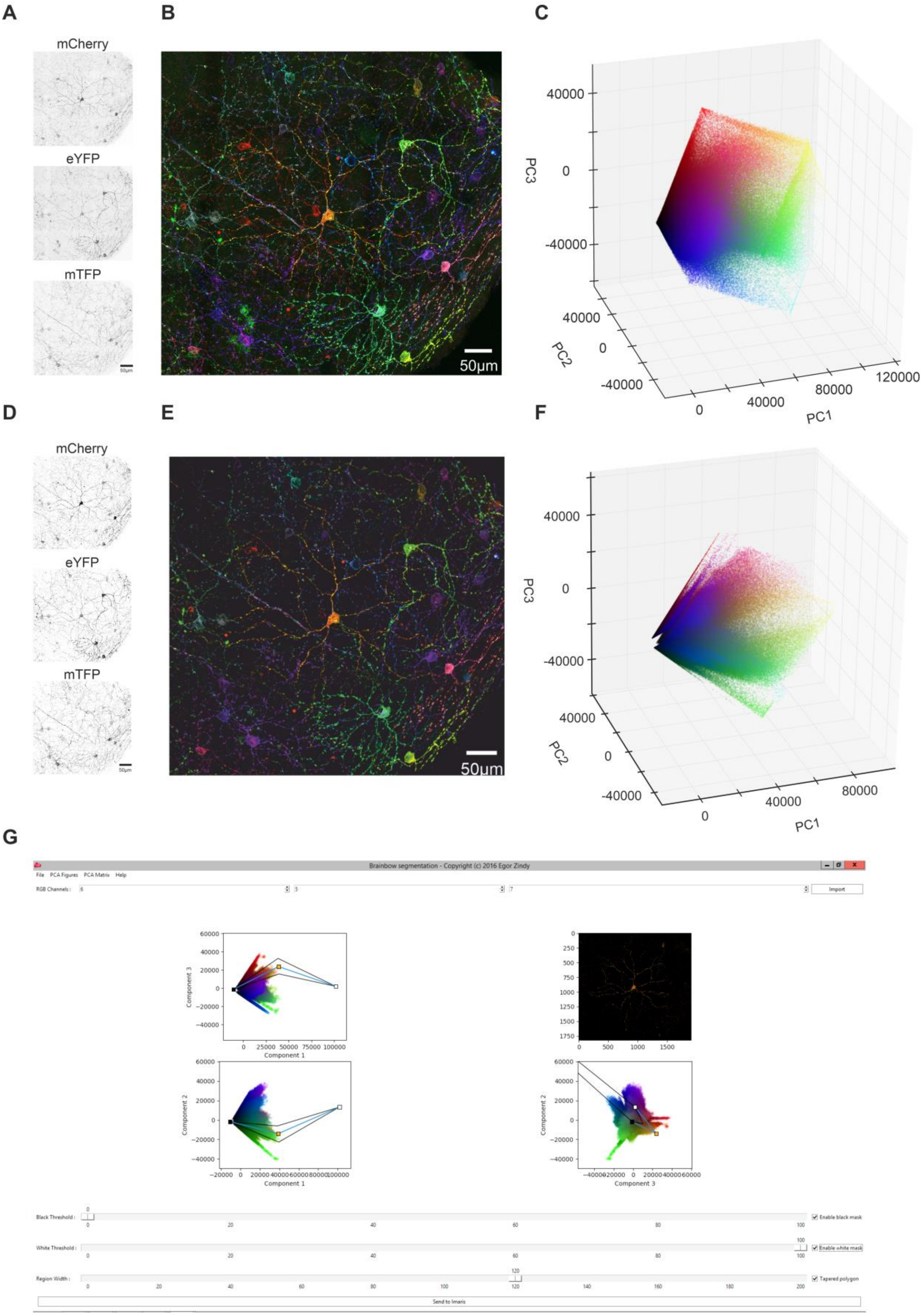

Supplementary Figure 2: Identifying and isolating voxel clusters from multi-colour images in Principal Component Space and Screenshot of the BRIAN GUI. *Unfiltered (A-B) and filtered (D-E) images from the Brainbow labelled Opn4*$^{Cre/+}$ *retina (shown in Figure 1B and C) and the corresponding*

PCA 3D plots from unfiltered (C) and filtered (F) images showing more distinguishable voxels clusters. (G) 3 x 2D representation of PC space (PC1 vs. PC2, PC1 vs. PC3 and PC2 vs. PC3) of the ROI from the Opn4$^{Cre/+}$ retina in Figure 1C and 3A. There are three panels with 2D representations of the PC space (PC1 vs. PC2, PC1 vs. PC3 and PC2 vs. PC3) from the filtered ROI in Figure 3A. The orange cluster can be clearly visualised in 2 of the 3 representations of PC space (PC1 vs. PC3 & PC2 vs. PC3). 2D PCA plots are linked in PC space therefore the user only needs to identify and visualize a cluster in 2 x 2D PCA representations. The 'handle point' of a tapered polygon was placed at the furthest point away from the black point and encompassed the orange cluster of voxels. There is also a window for a live maximum projection of the spatial reconstruction of the polygon-selected voxels which demonstrated that the identified orange cluster represented the orange ipRGC in the original image. User can set the width of the tapered polygon at the sliding bar on the bottom.

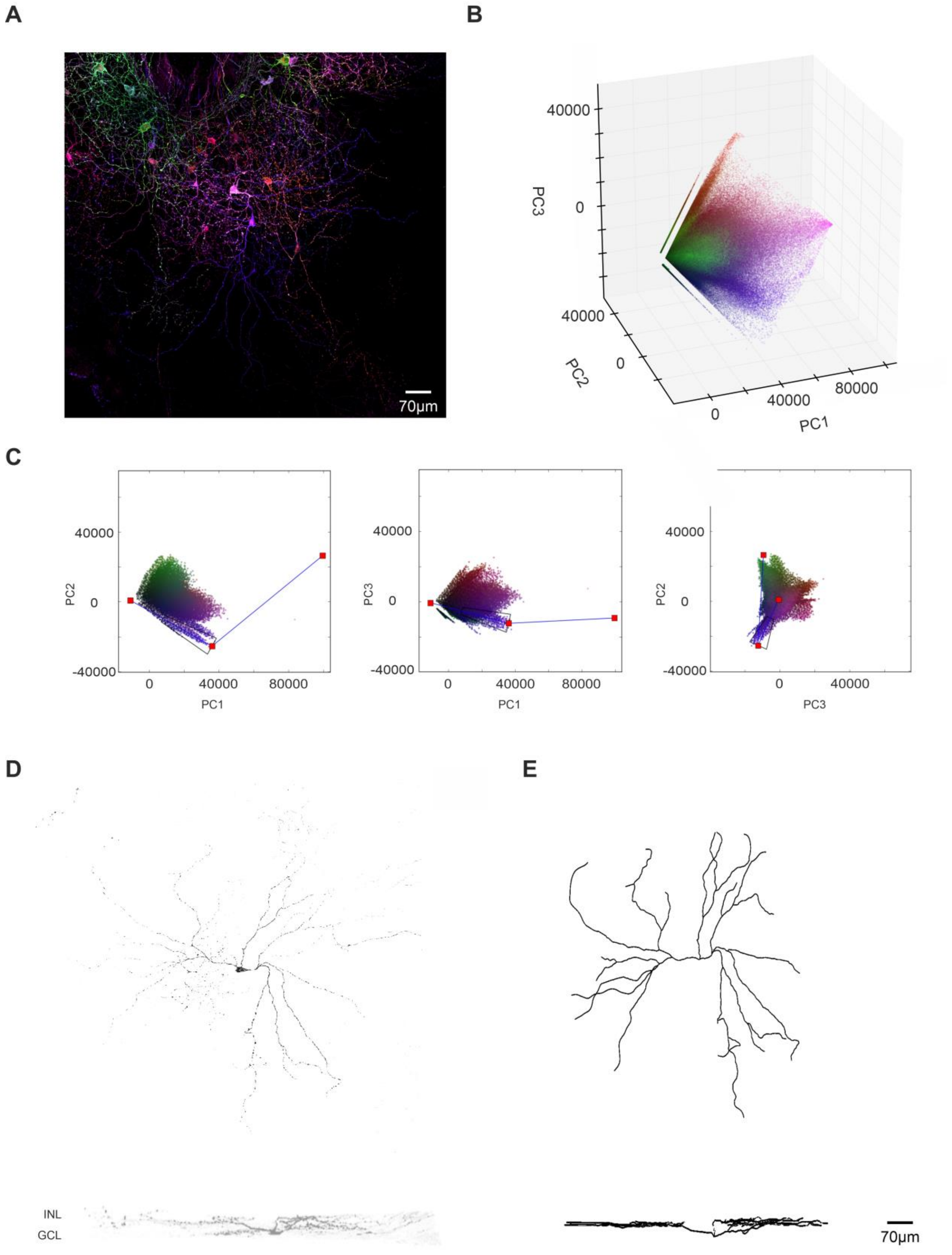

A
70μm
B
PC3
40000
0
40000
PC2
0
0
40000
80000
PC1
C
PC2
40000
0
-40000
0
40000
80000
PC1
PC3
40000
0
-40000
0
40000
80000
PC1
PC2
40000
0
-40000
0
40000
PC3
D
INL
GCL
E
70μm

Supplementary Figure 3: Identification of an uncategorised ipRGC in the Opn4$^{Cre/+}$;rd/rd retina.
(A) *Pseudocoloured immunofluorescence image of the ROI for following image pre-filtering to reduce background noise and remove voxels with a signal in only one channel (see Online Methods).* ***(B)*** *3D representation of PC space (PC1 vs. PC2 vs. PC3) of the ROI following image pre-filtering.* ***(C)*** *Following pre-filtering of the ROI an identifiable 'purple' cluster of voxels became apparent in PCA space, shown here in sequential 2D projections in PC space of randomly selected 80,000 voxels (a number which we found to give a good visual approximation of the clusters at reduced rendering time). A user defined trapezoid encompassing the voxels in this cluster is shown in black.* ***(D)*** *Spatial location of voxels in XY (upper) and XZ (lower) orientations of voxels from the ROI falling within the region of PCA space described by the trapezoid in (C) project an image consistent with that of a single cell in XY and XZ dimensions and its reconstruction using filament tracer, IMARIS* ***(E).*** *Locations of inner nuclear layer (INL) and ganglion cell layer (GCL) provided as references for Z-projection images.*

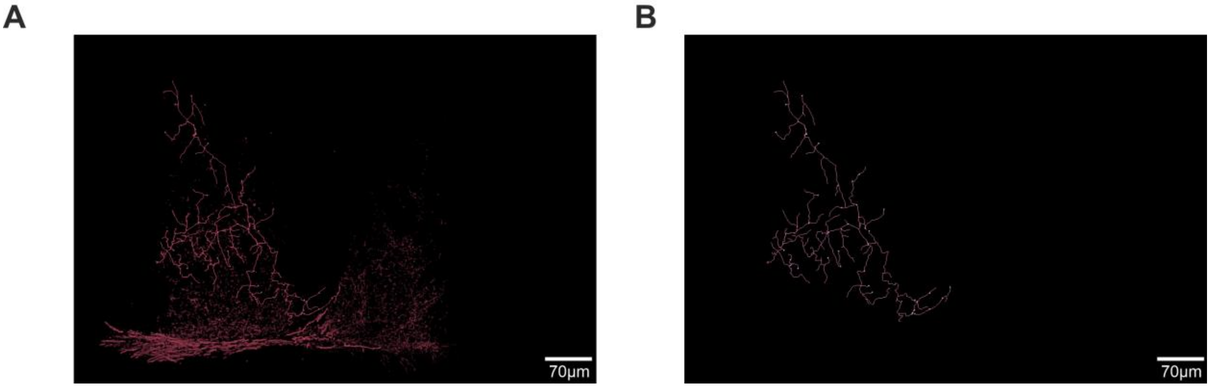

Supplementary Figure 4: A single axonal projection of the SCN identified using BRIAN. (A) *Spatial reconstruction of an axonal projection of a single neuron in the SCN, which was continuously traced from the red cluster shown in Figure 7.* ***(B)*** *Filament tracer of the single axonal projection from (A).*

	Cell #	Stratification	Soma size (µm)	Dendritic field diameter (µm)	Number of branch points	Fraction of total	Reference
M1	1	OFF	13.82	221.34	7.00		
	2	OFF	13.68	209.46	8.00		
	3	OFF	10.30	243.02	6.00		
	4	OFF	12.46	235.00	12.00		
Retinally Degenerate	Mean ± S.E.M		12.6± 0.8	227.2 ± 7.4	8.3 ± 1.3	4/35	
Visually Intact			13.9 ± 0.5	290.1 ± 16.5	10.2 ± 1.6	-	1
M2	5	ON	13.72	285.84	20.00		
	6	ON	15.10	309.46	14.00		
	7	ON	15.58	265.96	29.00		
	8	ON	15.81	252.28	37.00		
	9	ON	14.10	301.92	18.00		
	10	ON	14.98	358.08	28.00		
	11	ON	16.26	287.42	19.00		
	12	ON	15.94	279.82	38.00		
	13	ON	15.24	266.62	31.00		
	14	ON	15.54	268.76	38.00		
Retinally Degenerate	Mean ± S.E.M		15.2 ± 0.3	287.6 ± 9.6	27.2 ± 2.8	10/35	
Visually Intact			15.7 ± 0.4	316.6 ± 13.8	24.4 ± 1.5	-	1
M3	15	Bistratified	18.50	228.12	44.00	1/35	
Visually Intact	Mean ± S.E.M		17.8 ± 0.6	477.4 ± 20.1	*Not Reported*	-	2
M4	16	ON	19.16	247.78	43.00		
	17	ON	18.38	325.26	52.00		
	18	ON	21.58	315.58	39.00		
	19	ON	19.7	282.58	40.00		
	20	ON	20.68	323.74	45.00		
	21	ON	19.75	280.90	61.00		
	22	ON	17.26	277.46	47.00		
	23	ON	18.62	275.94	43.00		
	24	ON	22.37	331.86	43.00		
	25	ON	20.90	297.88	48.00		
	26	ON	20.41	325.74	40.00		
Retinally Degenerate	Mean ± S.E.M		19.9 ± 0.5	298.6 ± 8.3	45.5 ± 1.9	11/35	
Visually Intact			21.0 ± 0.4	359.6 ± 12.8	38.2 ± 1.6	-	1
M5	27	ON	14.40	177.26	32.00		
	28	ON	15.52	200.42	42.00		
	29	ON	14.40	192.98	29.00		
	30	ON	14.86	159.72	33.00		
	31	ON	12.90	220.06	39.00		
	32	ON	16.46	215.26	41.00		
	33	ON	13.68	214.48	32.00		
Retinally Degenerate	Mean ± S.E.M		14.6 ± 0.4	197.2 ± 8.4	35.4 ± 1.9	7/35	
Visually Intact			14.2 ± 2.4	223.7 ± 43.9	52.1 ± 12.5	-	- 3
M6	34	Bistratified	13.90	239	41.00	1/35	
Visually Intact	Mean ± S.E.M		12.7 ± 1.8	216 ± 30	100 ± 27	-	4
Uncategorised		-	-	-	-	-	
Retinally Degenerate	35	OFF	13.80	546.00	21.00	1/35	

[1] *Ecker et al (2010), Melanopsin-expressing retinal ganglion cell photoreceptors: Cellular diversity and role in pattern vision*

[2] *Schmidt & Kofuji (2011), Structure and function of the bristratified intrinsically photosensntive ganglion cells in the mouse*

[3] *Stabio et al (2018), The M5 Cell: A Colour-Opponent Intrinsically Photosensitive Retinal Ganglion Cell*

[4] *Quattrochi et al (2019), The M6 cell: A small-field bistratified photosensitive retinal ganglion cell*

***Supplementary Table 1**: **Quantitative morphological analysis of ipRGCs in the $Opn4^{Cre/+}$;rd/rd mouse using BRIAN**. (A) Data from individual cells for soma size, dendritic field diameter and total number of branch points for the six reported ipRGC subtypes identified from four $Opn4^{Cre/+}$;rd/rd retinas using the BRIAN platform. Single cell data was collected using the commercially available plug-in Filament Tracer (IMARIS, Bitplane). Visually intact data collected from published literature on $Opn4^{Cre/+}$ mice and previously reported.*

Supplementary Video 1: 3D representation of close up of a representative rob bipolar cell shown in Figure 2G*. The video starts with the original imaged volume, following with filtering using BRIAN. Then it shows voxels from one purple cluster isolated using BRIAN, and then traced using Filament Tracer (Imaris) revealing a rod bipolar cell based on morphology of the reconstructed cell.*

Supplementary Video 2: 3D representation of close up of a representative cone bipolar cell shown in Figure 2H*. The video starts with the original imaged volume, following with filtering using BRIAN. Then it shows voxels from one yellow cluster isolated using BRIAN, and then traced using Filament Tracer (Imaris) revealing a cone bipolar cell based on morphology of the reconstructed cell.*

Supplementary Video 3: 3D representation of SCN shown in Figure 7*. The video starts with the original imaged volume, following with filtering using BRIAN. Then it shows one red cluster isolated using BRIAN, and then traced using Filament Tracer (Imaris). Finally, it shows all the 7 clusters we isolated and their traced filaments.*
